# Reduced classical probiotic-associated butyrate biosynthetic potential predicts early primary resistance to anti-PD-1 therapy in advanced non-small cell lung cancer: a shotgun metagenomic study

**DOI:** 10.3389/fcimb.2026.1975315

**Published:** 2026-10-28

**Authors:** Yaoguo Mei, Ling Shi, Sunxiang He, Chaoxing Liu, Meifen Liu, Yanjun Hou

**Affiliations:** 1 Department of Oncology, Jiujiang Hospital of Traditional Chinese Medicine, Jiujiang, Jiangxi, China; 2 Department of Oncology, First People’s Hospital of Jiujiang City, Jiujiang, Jiangxi, China; 3 Department of Oncology, The First Affiliated Hospital, Jiangxi Medical College, Nanchang, Jiangxi, China

**Keywords:** anti-PD-1 therapy, classical probiotic–butyrate axis, early primary resistance, non-small cell lung cancer, probiotic-associated ecology

## Abstract

**Background:**

Early primary resistance to anti-PD-1 therapy is common in advanced non-small cell lung cancer (NSCLC). Existing microbiome biomarkers largely rely on individual species and lack stability across populations.

**Objective:**

The aim of this study was to construct the classical probiotic–butyrate axis score (PBAS), perform external validation, and evaluate its value for risk stratification of 90-day early primary resistance and PFS.

**Methods:**

This study adopted a dual-cohort, patient-level secondary analysis design and reanalyzed pretreatment fecal shotgun metagenomic data from two public cohorts, including 70 and 40 patients in the discovery and external validation cohorts, respectively. An equally weighted PBAS was constructed based on predefined classical probiotic-associated taxa and the butyryl-CoA:acetate CoA-transferase (*but*) and the butyrate kinase (*buk*) genes. ANCOM-BC2 was used for differential analysis, Firth logistic regression for association analysis of early primary resistance, 2,000 patient-level bootstrap resampling for internal validation, and Cox proportional hazards models for PFS analysis.

**Results:**

Early primary resistance occurred in 41/70 and 23/40 patients in the two cohorts, respectively. Classical probiotic-associated taxa, the butyrate functional subscore, and PBAS were lower in the resistant group than in the nonresistant group, whereas *buk* and *Akkermansia muciniphila* showed no significant differences. After adjustment for age, sex, and BMI, acetyl-CoA pathway potential was also reduced. For each 1-SD increase in PBAS, the OR for early primary resistance was 0.39 and 0.37 after adjustment for clinical variables. The bootstrap-corrected area under the receiver operating characteristic curve (AUC) was 0.733, and the external validation AUC was 0.726 (95% CI: 0.559–0.894). Increased PBAS was associated with a lower risk of progression or death, with HRs of 0.54 and 0.61 in the two cohorts, respectively (both *p* < 0.001).

**Conclusion:**

Combined reductions in pretreatment classical probiotic-associated ecology and butyrate biosynthetic potential indicate a high risk of early primary resistance to anti-PD-1 therapy in advanced NSCLC and support PBAS as an exploratory outcome risk marker for cross-cohort risk stratification among patients receiving anti-PD-1 therapy, without establishing treatment-specific predictive value.

## 1 Introduction

Lung cancer remains one of the leading causes of cancer incidence and mortality worldwide, with nearly 2.5 million new cases and more than 1.8 million deaths in 2022 (Bray et al., 2024. Non-small cell lung cancer constitutes the majority of lung cancers, and anti-PD-1 immunotherapy has become an important treatment for patients with advanced disease. However, a substantial proportion of patients develop primary resistance early during treatment and have difficulty achieving sustained benefit (Thai et al., 2021; Passaro et al., 2022). Although Programmed Death-Ligand 1 (PD-L1) expression, tumor mutation burden, and conventional clinical factors can assist in risk stratification, they cannot fully explain interindividual differences (Mino-Kenudson et al., 2022). The gut microbiota can influence antitumor immunity through the mucosal barrier, antigen presentation, T-cell activation, and systemic metabolic regulation, and its composition and metabolic functions have also been associated with the efficacy of immune checkpoint inhibitors (Villemin et al., 2023). Previous studies have observed enrichment of *Akkermansia muciniphila*, *Bifidobacterium*, and certain butyrate-producing bacteria in responders, making pretreatment fecal metagenomic data a potentially valuable source of noninvasive risk information (Thomas et al., 2023).

Current evidence still mainly focuses on individual response-associated species, and signals from dominant bacterial taxa lack consistent stability across different regions and cohorts Lee et al., 2022). Taxonomic differences are readily influenced by diet, medications, sequencing platforms, and background microbiota, and single-species models also have difficulty reflecting metabolic cooperation within microbial communities (Elkrief et al., 2025). Classical probiotic-associated taxa can produce acetate and lactate and support direct butyrate-producing bacteria, while butyrate may participate in maintaining the intestinal barrier, regulating histone deacetylation, and enhancing cytotoxic T-cell function (Singh et al., 2023). Here, “probiotic-associated taxa” denotes only metagenomically detected taxonomic signals and does not imply probiotic intake, strain colonization, or *in vivo* functional activity. Animal and translational studies have suggested that *Bifidobacterium* and butyrate can improve some responses to PD-1 or PD-L1 blockade (Kang et al., 2024). In advanced non-small cell lung cancer (NSCLC), whether classical probiotic-associated ecological features and butyrate biosynthetic potential are jointly reduced and whether they can stably identify early primary resistance remain insufficiently investigated (Jin et al., 2025). Previous studies have also frequently interpreted species abundance, metabolic gene potential, and actual metabolite levels together, failing to distinguish microbial coding capacity from actual butyrate exposure (Valles-Colomer et al., 2023). After excluding the repeatedly studied *A. muciniphila*, a transparent score that integrates taxonomic and functional information and can be validated using a locked model across cohorts has not yet been established.

In this study, pretreatment fecal shotgun metagenomic data from CNP0000636 and PRJEB22863 were uniformly reanalyzed, classical probiotic-associated taxa and the butyryl-CoA:acetate CoA-transferase (*but*) and the butyrate kinase (*buk*) genes were predefined, and the classical probiotic–butyrate axis score was constructed and validated in an independent NSCLC cohort. The taxonomic and functional components represent complementary aspects of the same ecological–functional axis, and therefore they were standardized and assigned equal weights *a priori* to avoid outcome-driven weighting and reduce overfitting in the small development cohort. The study evaluated the association of this score with 90-day early primary resistance, as well as its discrimination, calibration, and clinical net benefit, and examined its robustness and temporal stability using PFS and short-term longitudinal samples.

## 2 Materials and methods

### 2.1 Study design

This study adopted a dual-cohort, patient-level secondary analysis design using pretreatment fecal shotgun metagenomic data. CNP0000636 (Fang et al., 2022) served as the discovery and model-development cohort, with raw data downloaded from the CNGB Sequence Archive; PRJEB22863 (Routy et al., 2018) served as the independent external validation cohort, with raw data downloaded from the European Nucleotide Archive. The original study of the discovery cohort included 85 patients with advanced NSCLC. After excluding patients with antibiotic exposure, 285 longitudinal fecal samples from 73 patients were included in the shotgun metagenomic analysis; only M0 samples collected before the first treatment administration were used in the primary analysis. The external project registered 219 samples, 219 experiments, and 219 runs, covering NSCLC and renal cell carcinoma; this study extracted only pretreatment T0 samples from patients with NSCLC and permanently excluded renal cell carcinoma and other tumor samples. All raw Sequence and quality information format (FASTQ) files were reanalyzed using the same workflow. The two cohorts were not pooled for training, no cross-cohort batch correction was performed, and external outcomes were not used to update the model. The unit of analysis was the patient, and repeated samples from the same patient or multiple sequencing runs from the same fecal sample were not treated as independent observations. Outcomes in the external cohort remained blinded until sample selection, the feature dictionary, the scoring formula, and the analysis code had been frozen.

A four-level mapping table linking patients, fecal samples, BioSamples, and runs was established, and tumor type, sampling time, time of first anti-PD-1 administration, antibiotic exposure, efficacy assessment, and progression-free survival (PFS) were integrated. Multiple lanes or runs from the same physical sample were merged at the FASTQ level; when multiple pretreatment samples were available from the same patient, the sample closest to the first treatment administration was selected; when sampling dates were identical, the sample with the higher number of valid reads after host depletion was selected. Records for which unique mapping could not be completed were excluded. A unique reason was recorded for every inclusion, exclusion, and deduplication decision, and an auditable sample-processing log was retained.

### 2.2 Study population, baseline samples, and outcome definitions

Patients were included if they were aged at least 18 years, had histologically or cytologically confirmed advanced or recurrent NSCLC, received anti-PD-1 monoclonal antibody-containing therapy, and had an identifiable pretreatment fecal sample and an assessable 90-day clinical outcome. Patients with peritreatment antibiotic exposure as defined in the original studies, those with only posttreatment samples, those with indeterminate outcomes, those with unclear sample identities, and those who failed technical quality control were excluded. The external cohort followed the antibiotic definition used in the original project, namely, receipt of systemic antibacterial agents from 2 months before to 1 month after the first immunotherapy administration. Patients with RCC were not included in the primary analysis, and posttreatment T1 samples were not used to replace missing T0 samples.

The primary outcome was study-defined 90-day early primary resistance. Patients with PFS no longer than 90 days, a best overall response of progressive disease (PD) or fast death (FD), or radiographic progression or death within 90 days were classified into the resistant group; patients with a best overall response of complete response, partial response, or stable disease and PFS longer than 90 days were classified into the nonresistant group. Patients who remained progression-free before 90 days but were lost to follow-up, whose efficacy assessment and PFS were mutually inconsistent and could not be verified, or for whom sufficient information was unavailable were not included in the primary outcome analysis. PFS was calculated from the date of first anti-PD-1 administration to radiographic progression or death, and patients without an event were censored at the date of the last confirmed progression-free assessment.

### 2.3 Raw metagenomic sequencing data preprocessing and quality control

The original FASTQ files were first checked for file size and MD5 values. FASTQ preprocessing and quality-control tool (fastp) was used to remove adapters and poly-G tails, trim low-quality bases from both ends, with the quality threshold set at Q20; reads containing more than five ambiguous bases and reads shorter than 35 bp after trimming were removed. Both cohorts were processed using a single-end library structure. The cleaned reads were aligned to GRCh38.p14 using Bowtie2 in very-sensitive mode; reads aligned to the human genome were removed. Samples with fewer than 5 million valid reads after host depletion, corrupted FASTQ files, failure of the species annotation workflow, or failure of the Integrated Gene Catalog (IGC) quantification workflow were excluded. For each sample, the number of raw reads, number of cleaned reads, proportions of Q20 and Q30 bases, host-read proportion, number of nonhost reads, bacterial classification rate, and IGC mapping rate were recorded. Technical exclusions were completed before linkage to clinical outcomes. No rarefaction subsampling was performed throughout the workflow, and no differential quality-control thresholds were set according to outcome.

### 2.4 Species-level taxonomic analysis

Kraken2 was used for read-level classification, and Bracken was used for correction at the species level. The same frozen version of the RefSeq taxonomic database was used for both cohorts, and corresponding Bracken read-length distribution files were generated for the BGISEQ-500 SE50 reads in the discovery cohort and the Ion Proton single-end reads in the external cohort, respectively. Species relative abundance was calculated using the bacterial reads estimated by Bracken as the denominator, and undetected species were coded as 0. Taxonomic names were harmonized according to NCBI taxon ID, and the broad lactobacilli group was merged using a predefined and frozen old-name-to-new-name taxon ID dictionary to avoid duplicate counting caused by taxonomic revisions. Exploratory species-level differential analysis included only species detected in at least 10% of patients.

### 2.5 Analysis of butyrate biosynthetic functional potential

Host-depleted reads were uniformly aligned to the human gut microbial 9.9 M integrated gene catalog. Records with an alignment identity of at least 95% and an effective alignment length of at least 35 bp were retained; multiple equally best matches were proportionally allocated, and gene counts were calculated using single-end reads as the unit. Gene counts were converted to TPM after length correction. The butyrate functional module consisted of the *but* and the *buk* (Vital et al., 2014). The *but* gene was identified from IGC-predicted protein sequences using validated reference sequences and hidden Markov models, and not all *K01034* genes were directly classified as *but*; *buk* was identified based on *K00929* and EC 2.7.2.7. Both gene types were required to have an HMM *E*-value of no greater than 1 × 10^−5^ and target-domain coverage of at least 70%; however, candidate genes were additionally reviewed on the basis of EC 2.8.3.8 and phylogenetic position. The target gene list was frozen before linkage to resistance outcomes. Target genes taxonomically annotated as originating from *Akkermansia muciniphila* were removed from the functional module. The four butyrate biosynthesis pathways involving acetyl-CoA, glutarate, 4-aminobutyrate, and lysine were used only for mechanistic interpretation and were not included in the score.

### 2.6 Construction of the classical probiotic–butyrate axis score

The classical probiotic-associated taxonomic module was predefined as the genus *Bifidobacterium*, the broad lactobacilli group, *Streptococcus thermophilus*, and *Clostridium butyricum* (Guarner et al., 2024). These signals represented only probiotic-associated taxa detected by metagenomics and were not used to infer the intake or stable colonization of commercial strains. *A. muciniphila* was permanently excluded from the score and was used only as an independent comparator feature; its abundance was transformed as log_10_(1 + 10^6^ × relative abundance).

Let *R* denote the total relative abundance of classical probiotic-associated taxa in a patient, and the taxonomic subscore was defined as *T* = log_10_(1 + 10^6^*R*); let *B* denote the sum of the abundances of the *but* and *buk* genes, and the functional subscore was defined as *F* = log_10_(1 + *B*). *T* and *F* were both converted to Z-scores using the means and standard deviations of the baseline samples in the discovery cohort, and were combined with equal weights as the probiotic–butyrate axis score (PBAS)^*^ = (ZT + ZF)/2. PBAS^*^ was then standardized using its mean and standard deviation in the discovery cohort to obtain the classical PBAS. A higher PBAS indicated a stronger associated ecological module and greater genetic potential for butyrate biosynthesis. The external cohort directly used the locked species dictionary, gene set, means, standard deviations, and scoring formula from the discovery cohort, without reweighting or restandardization. PBAS was treated as a continuous variable in the primary analysis; only for presentation of the survival curves was the median of the discovery cohort used to define the high and low groups, and the same threshold was applied to the external cohort. Features were not added or removed, weights were not adjusted, and thresholds were not optimized during score construction based on *p*-values from differential analyses, area under the receiver operating characteristic curve (AUC), or external validation results.

### 2.7 Statistical analysis of microbiome features

Continuous clinical variables were described using medians and interquartile ranges, and categorical variables were described using counts and percentages. Balance between cohorts and outcome groups was evaluated using standardized differences, with an absolute standardized difference of no less than 0.10 indicating distributional imbalance, although this criterion was not used to select adjustment variables. All eligible patients in the two datasets were included, and no *post hoc* power analysis was performed. The primary outcome, PBAS, and its component variables were not imputed. When the missing proportion of a clinical covariate was 20% or less, multiple imputation by chained equations was performed. The imputation model included the outcome, PBAS, and all prespecified covariates, generated 20 datasets, and pooled the results according to Rubin’s rules; variables with more than 20% missing data were not included in the multivariable model, and a complete-case sensitivity analysis was also performed.

Between-group differences in the predefined taxonomic module, *but*, *buk*, the butyrate functional module, and PBAS were assessed using the Wilcoxon rank-sum test. Differential analyses of all species and functional features were performed using Analysis of Compositions of Microbiomes with Bias Correction 2 (ANCOM-BC2), with models adjusted for age, sex, and body mass index. This parsimonious adjustment set was used to limit overparameterization in the high-dimensional exploratory analyses of the small cohorts, whereas smoking status, histology, and treatment regimen were additionally included in the low-dimensional PBAS outcome models and complete-case sensitivity analysis. The Benjamini–Hochberg method was used to control the false discovery rate, and *q* < 0.05 was considered exploratorily significant. The association between the taxonomic subscore and the functional subscore was assessed using Spearman correlation analysis; the species origins of butyrate genes were summarized at the species and genus levels according to the best IGC annotations. No mediation analysis was performed.

### 2.8 Establishment and validation of the prediction model for early primary resistance

The primary association and prediction models used Firth bias-reduced logistic regression, with the model specified as logit[*p*(early primary resistance)] = *α* + *β* × PBAS. Effect sizes were reported as odds ratios and 95% confidence intervals per 1 discovery-cohort SD increase in PBAS. The prespecified adjusted model included continuous age, sex, continuous body mass index, smoking status (never/former or current), histological type (squamous/nonsquamous), and treatment regimen (anti-PD-1 monotherapy/anti-PD-1-containing combination therapy). These covariates were selected *a priori* based on clinical relevance, independently of observed outcome associations and the final complete-case count, with inclusion governed by the prespecified missing-data rule. Given the limited event-to-predictor ratio in the seven-predictor clinical + PBAS model, it was treated as an adjusted association and comparator model and interpreted cautiously rather than as the primary prediction model. The main model used Firth intercept correction to generate individual predicted probabilities. Comparator models separately included the taxonomic subscore, functional subscore, *A. muciniphila* abundance, PBAS combined with *A. muciniphila*, clinical variables, and clinical variables combined with PBAS. LASSO, random forest, sample splitting, oversampling, and synthetic cases were not used.

Internal validation was performed using 2,000 patient-level bootstrap repetitions. In each resample, the standardization parameters, PBAS, and model coefficients were recalculated, and apparent performance and optimism were estimated. The optimism-corrected AUC, calibration intercept, calibration slope, and Brier score were reported. External validation was conducted once after the feature dictionary, scoring parameters, model coefficients, and code had been frozen, without refitting or recalibration. For the external cohort, the DeLong AUC and 95% confidence interval, calibration intercept, calibration slope, Brier score, and a calibration plot based on quartiles of predicted risk were reported. Decision curve analysis calculated net benefit across threshold probabilities of 0.20–0.80 and compared it with the classify-all-as-high-risk and classify-all-as-low-risk strategies. Differences in AUC were assessed using the DeLong test. All tests were two-sided, with a significance level of 0.05.

### 2.9 Secondary outcomes and robustness analyses

PFS was analyzed using Cox proportional hazards models, and hazard ratios and 95% confidence intervals per 1-SD increase in PBAS were reported. The proportional hazards assumption was tested using Schoenfeld residuals. Kaplan–Meier curves were stratified according to the median PBAS in the discovery cohort, and the same threshold was applied to the external cohort. Between-group comparisons were performed using the log-rank test. The full time-to-event PFS analysis was considered supportive rather than an independent validation because PFS ≤ 90 days contributed to the primary composite outcome.

Prespecified sensitivity analyses included defining resistance solely according to PFS ≤ 90 days, defining resistance solely according to RECIST response, adding *A. muciniphila* abundance to the model, restricting the analysis to patients receiving anti-PD-1 monotherapy, using complete-case data, and separately analyzing *but* and *buk*. The response-only analysis was prespecified to assess robustness independently of the PFS-based component of the primary outcome. Longitudinal samples collected within 30 days after treatment and before progression or death in the discovery cohort were used for the stability analysis, using a linear mixed-effects model with a patient-level random intercept: PBAS ~ time + resistance status + time × resistance status + (1|patient). Longitudinal samples were not included in the primary prediction model and did not increase the number of evaluable patients.

### 2.10 Software environment and study reproducibility

The data-processing workflow was uniformly orchestrated using Snakemake. The main software included fastp 0.23.4, Bowtie2 2.5.4, Kraken2 2.1.3, Bracken 2.9, and HMMER 3.4. Statistical analyses were performed in R 4.4.1 using the ANCOM-BC, logistf, pROC, rms, rmda, survival, lme4, and mice packages. Version numbers and MD5 values were recorded for the software, databases, container images, reference files, and feature dictionary; fixed random seeds were set for all random processes. External outcomes remained blinded until the model and code had been locked, and all intermediate files, parameters, exclusion logs, and analysis scripts were placed under version control.

### 2.11 Ethics statement and standardized reporting

This study used only publicly available, deidentified data and involved no additional participant recruitment, sample collection, or clinical intervention; therefore, ethics review and renewed informed consent could be waived. This study was reported in accordance with the Strengthening The Organization and Reporting of Microbiome Studies (STORMS) and Transparent Reporting of a Multivariable Prediction Model for Individual Prognosis or Diagnosis (TRIPOD)+AI guidelines (see the **Supplementary Files** STORMS_Checklist and TRIPOD_AI_Checklist, respectively), and the risk of bias was assessed using Prediction Model Risk of Bias Assessment Tool + Artificial Intelligence (PROBAST+AI).

## 3 Results

### 3.1 Study population, clinical outcomes, and metagenomic data quality

After patient-level screening, a baseline analysis set of 70 patients was established from 73 patients and 285 samples in the discovery cohort, of whom 49 were included in the 30-day longitudinal analysis; a baseline validation set of 40 patients with NSCLC was selected from 100 patients and 219 samples in the external cohort. The model was locked after internal validation and directly applied to independent external validation (**Figure 1**). Patient age, sex, smoking history, and histological composition were generally similar between the two cohorts. Patients in the resistant group were older and had lower BMI, with within-cohort |SMD| values of 0.420–0.432 and 0.453–0.539, respectively. The proportion of patients receiving combination therapy was lower in the external cohort than in the discovery cohort (10.00% vs. 32.86%, |SMD| = 0.580) (**Table 1**). The 90-day outcomes were determined for all patients in both cohorts. Early primary resistance occurred in 41/70 patients (58.57%) and 23/40 patients (57.50%), respectively, and the PFS event rates during the entire follow-up period were 88.57% and 90.00%, respectively; the median PFS was 2.86 and 2.89 months, respectively (**Table 2**). A total of 110 pretreatment fecal samples from the two cohorts passed the prespecified quality-control criteria. Sequencing depth was higher in the discovery cohort than in the external cohort, but the post-quality-control read retention rates exceeded 94% in both cohorts, with median numbers of valid nonhost reads of 79.80 × 10^6^ and 19.06 × 10^6^, respectively; the bacterial classification rates and IGC mapping rates remained high, and all analyzed samples met the prespecified analytical thresholds (**Table 3**). Sample-level quality control showed that 110 of the 112 candidate baseline samples met the prespecified criteria; one sample from each of the discovery and external cohorts was excluded because it contained fewer than 5 million valid nonhost reads. All remaining samples completed host depletion, species annotation, and IGC quantification, with no FASTQ corruption or analytical workflow failure (**Supplementary Table S1**).

**Figure 1:**
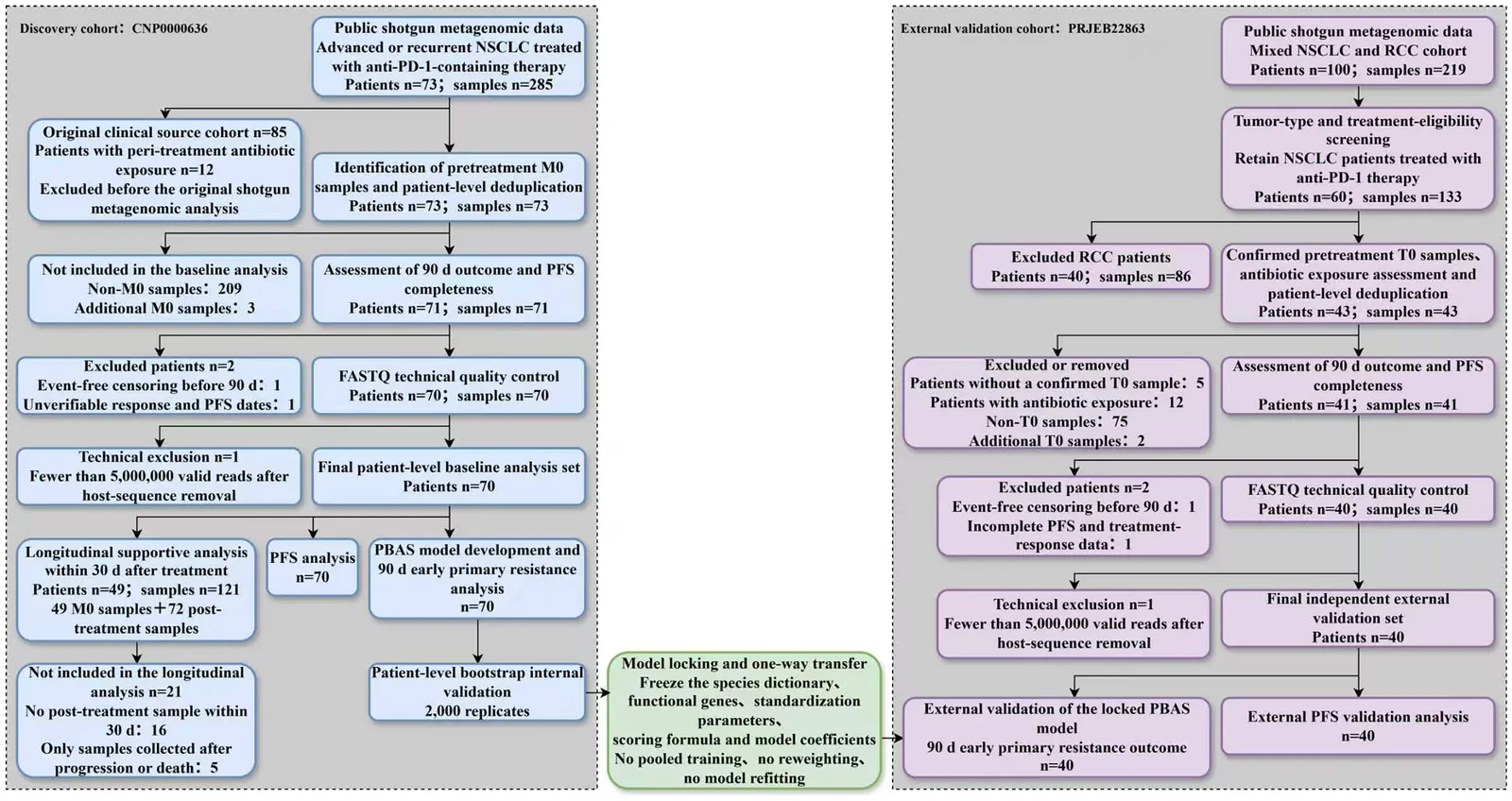
Study design and two-cohort analysis workflow. The primary unit of analysis was the patient, with one pretreatment baseline stool sample retained per patient. The discovery cohort was used for PBAS model development and internal validation, whereas the external cohort was used for independent validation of the locked model. *n*, number of patients; NSCLC, non-small cell lung cancer; RCC, renal cell carcinoma; M0/T0, pretreatment baseline time point; PFS, progression-free survival; PBAS, probiotic–butyrate axis score.

**Table 1:** Baseline characteristics of the discovery and external validation cohorts.

Characteristic	Discovery cohort				External validation cohort				Between-cohort |SMD|
	Overall (*n* = 70)	Early primary resistance (*n* = 41)	Nonresistant (*n* = 29)	Within-cohort |SMD|	Overall (*n* = 40)	Early primary resistance (*n* = 23)	Nonresistant (*n* = 17)	Within-cohort |SMD|	
Age (years)	64.5 (59.0, 69.0)	65.0 (61.0, 70.0)	61.0 (57.0, 68.0)	0.432	64.5 (60.0, 70.2)	65.0 (61.5, 71.0)	62.0 (60.0, 66.0)	0.420	0.122
Male sex (*n*/*N*; %)	55/70 (78.57)	32/41 (78.05)	23/29 (79.31)	0.031	31/40 (77.50)	18/23 (78.26)	13/17 (76.47)	0.043	0.026
BMI (kg/m^2^)	22.9 (22.1, 24.9)	22.6 (21.7, 24.7)	23.7 (22.3, 25.5)	0.453	24.2 (22.2, 26.1)	23.8 (22.1, 25.1)	25.3 (22.3, 26.5)	0.539	0.448
Former or current smoker (*n*/*N*; %)	51/68 (75.00)	31/40 (77.50)	20/28 (71.43)	0.140	31/39 (79.49)	18/23 (78.26)	13/16 (81.25)	0.074	0.107
Squamous-cell carcinoma (*n*/*N*; %)	22/70 (31.43)	15/41 (36.59)	7/29 (24.14)	0.273	12/40 (30.00)	8/23 (34.78)	4/17 (23.53)	0.250	0.031
Anti-PD-1-containing combination therapy (*n*/*N*; %)	23/70 (32.86)	15/41 (36.59)	8/29 (27.59)	0.194	4/40 (10.00)	3/23 (13.04)	1/17 (5.88)	0.247	0.580

Data are presented as median (Q1, Q3) or *n*/*N* (%), with percentages calculated using the number of patients with nonmissing data for each variable as the denominator. SMDs for continuous and binary variables were calculated using the pooled standard deviation and pooled binomial variance, respectively, and are reported as absolute values. An |SMD| ≥ 0.10 indicates distributional imbalance but was not used for covariate selection. The between-cohort |SMD| was used to assess model transportability to the external validation population. Baseline characteristics were not subjected to hypothesis testing.

*BMI*, body mass index; *IQR*, interquartile range; *SMD*, standardized mean difference.

**Table 2:** Best overall response, 90-day early primary resistance, and PFS in the discovery and external validation cohorts.

Clinical outcome	Discovery cohort (*n* = 70)	External validation cohort (*n* = 40)
Patients with assessable 90-day primary outcome	70/70 (100.00)	40/40 (100.00)
90-Day early primary resistance	41/70 (58.57)	23/40 (57.50)
90-Day nonresistance	29/70 (41.43)	17/40 (42.50)
Patients with evaluable best overall response	69/70 (98.57)	39/40 (97.50)
Complete response (CR)	1/69 (1.45)	0/39 (0.00)
Partial response (PR)	15/69 (21.74)	8/39 (20.51)
Stable disease (SD)	21/69 (30.43)	12/39 (30.77)
Progressive disease (PD)	26/69 (37.68)	15/39 (38.46)
Fast death (FD)	6/69 (8.70)	4/39 (10.26)
PFS events within 90 days	41/70 (58.57)	23/40 (57.50)
PFS events during total follow-up	62/70 (88.57)	36/40 (90.00)
Censored for PFS	8/70 (11.43)	4/40 (10.00)
Median PFS (months; 95% CI)	2.86 (2.60, 3.88)	2.89 (2.53, 4.57)

Data are presented as *n*/*N* (%) unless otherwise indicated. Early primary resistance was defined as PD, FD, PFS ≤ 90 days, or radiographic progression or death within 90 days; nonresistance was defined as CR, PR, or SD with PFS > 90 days. Response percentages were based on evaluable patients. Median PFS and 95% CI were estimated using the Kaplan–Meier method and converted using 30.44 days/month. No between-cohort or between-response-category hypothesis testing was performed.

*NR*, not reached; *FD*, fast death (was retained as originally recorded; see Section 2.2 for the outcome definition; *CI*, confidence interval; *PFS*, progression-free survival.

**Table 3:** Pretreatment fecal metagenomic sequencing and quality control in the two cohorts.

Technical metric	Discovery cohort (*n* = 70)	External validation cohort (*n* = 40)
Time from stool collection to first anti-PD-1 dose (day)	− 2 (− 5, − 1)	− 9 (− 17, − 3)
Single-end sequencing samples (*n*/*N*; %)	70/70 (100.00)	40/40 (100.00)
Raw reads per sample (× 10^6^)	83.74 (68.29, 99.12)	20.63 (17.89, 23.46)
Reads retained after fastp per sample (× 10^6^)	80.86 (65.82, 95.93)	19.46 (16.77, 22.14)
Post-QC read retention rate (%)	96.58 (95.91, 97.22)	94.33 (92.84, 95.71)
Bases with Q20 quality (%)	98.61 (98.17, 98.97)	96.74 (95.52, 97.63)
Bases with Q30 quality (%)	94.38 (93.46, 95.21)	88.91 (86.37, 91.12)
Human host-read proportion (%)	1.31 (0.57, 2.86)	2.08 (0.91, 4.12)
Nonhost reads per sample (× 10^6^)	79.80 (64.74, 94.20)	19.06 (16.23, 21.42)
Kraken2 bacterial classification rate (%)	76.42 (71.68, 81.07)	81.56 (76.91, 85.84)
IGC gene-catalog mapping rate (%)	72.87 (67.93, 77.68)	75.64 (70.36, 80.19)

Data are presented as median (Q1, Q3) or *n*/*N* (%). Negative values indicate stool collection before the first anti-PD-1 dose. Each single-end sequence record was counted as one read. Host-read proportions were calculated using fastp-processed reads as the denominator, whereas bacterial classification and IGC mapping rates used posthost-depletion reads. All samples met the prespecified threshold of ≥ 5 million nonhost reads. Only descriptive statistics were reported.

*IGC*, integrated gene catalog; *IQR*, interquartile range; *Q20/Q30*, proportions of bases with Phred quality scores ≥ 20/≥ 30.

### 3.2 Pretreatment microbial features associated with early primary resistance and differences in PBAS

In both cohorts, the total abundance of classical probiotic-associated taxa, *but* gene abundance, butyrate functional subscore, and PBAS were significantly lower in the resistant group than in the nonresistant group (all within-cohort comparisons, *p* < 0.05); no significant between-group differences in *buk* gene abundance or *A. muciniphila* abundance were observed in either cohort (all *p* ≥ 0.05) (**Figures 2A–F**). In the species-level ANCOM-BC2 analysis, after adjustment for age, sex, and BMI, the abundances of *Bifidobacterium longum*, *Clostridium butyricum*, *Faecalibacterium prausnitzii*, and *Agathobacter rectalis* were reduced in the resistant group, whereas *Enterococcus faecium* was increased (all *q* < 0.05); no significant difference was observed for *Akkermansia muciniphila* (**Supplementary Table S2**). In the ANCOM-BC2 analysis of the prespecified butyrate-related functional units, after adjustment for age, sex, and BMI, the abundances of *but*, thl, hbd, and cro were reduced in the resistant group and reached statistical significance in both cohorts (all *q* < 0.05); no significant differences in *buk* or the functional units of the 4-aminobutyrate and lysine pathways were observed in either cohort (**Supplementary Table S3**).

**Figure 2:**
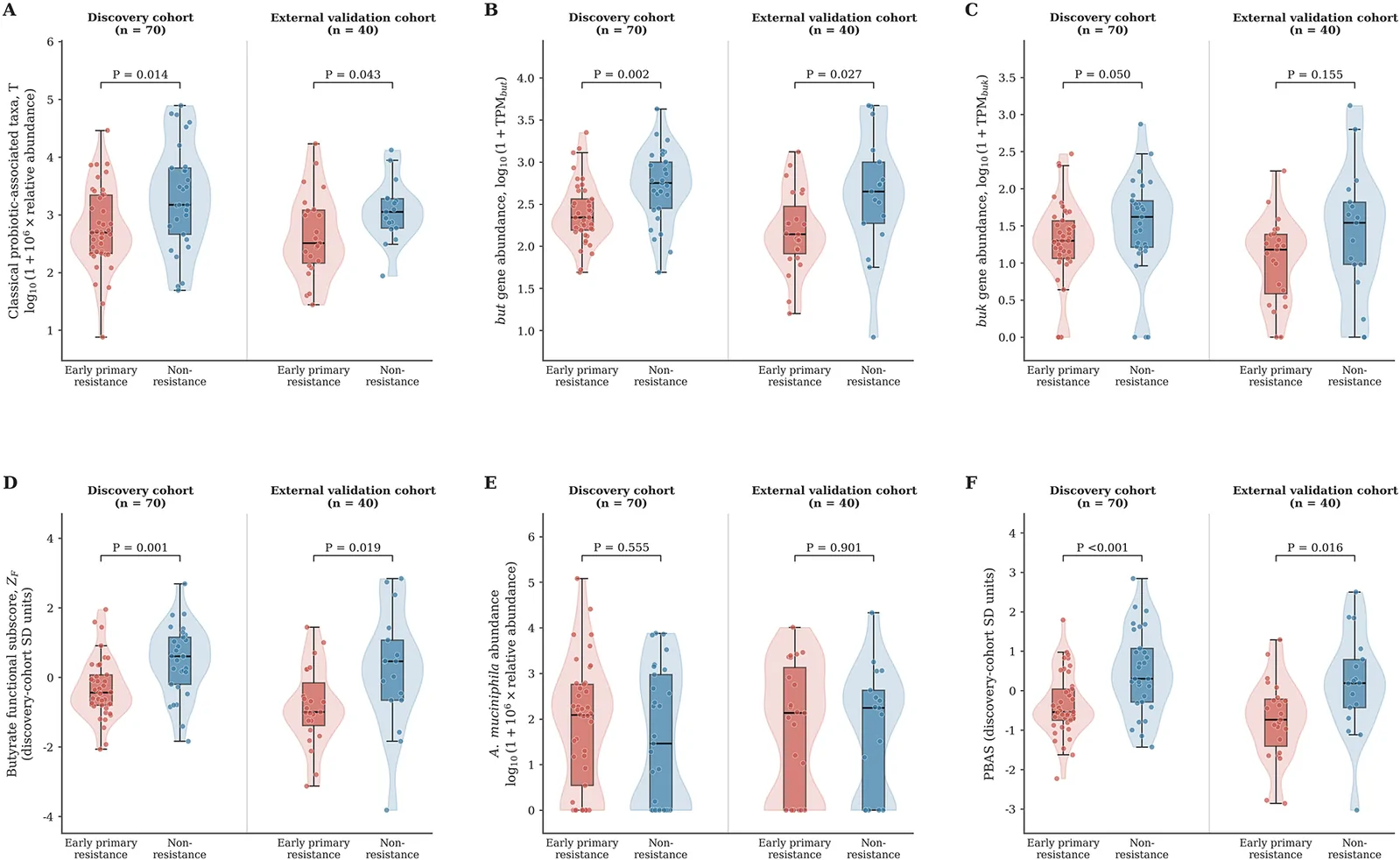
Distribution of pretreatment PBAS-related microbial features by early primary resistance status in the two cohorts. **(A)** Total abundance of classical probiotic-associated taxa (T); **(B)** *but* gene abundance; **(C)** *buk* gene abundance; **(D)** butyrate functional subscore (ZF); **(E)** *A. muciniphila* abundance; and **(F)** probiotic–butyrate axis score (PBAS). The discovery cohort included 70 patients (41 resistant and 29 nonresistant), and the external validation cohort included 40 patients (23 resistant and 17 nonresistant). Between-group comparisons were performed using two-sided Wilcoxon rank-sum tests with tie correction. PBAS was the primary measure; the remaining measures were supportive, and *p*-values were not adjusted for FDR. Undetected taxa or genes were coded as 0 before log_10_(1 + *x*) transformation. ZF and PBAS values in the external validation cohort were calculated using the locked discovery-cohort parameters. *but*, butyryl-CoA:acetate CoA-transferase gene; *buk*, butyrate kinase gene; PBAS, probiotic–butyrate axis score; TPM, transcripts per million; ZF, butyrate functional subscore.

### 3.3 Ecological-functional consistency of the classical probiotic–butyrate axis

The classical probiotic-associated taxonomic subscore was positively correlated with the butyrate functional subscore in both the discovery and external validation cohorts (*ρ* = 0.348, *p* = 0.003 and *ρ* = 0.532, *p* < 0.001, respectively) (**Figures 3A, B**). Among the individual species shown, the butyrate increase was mainly derived from *Faecalibacterium prausnitzii*, *Agathobacter rectalis*, and *Roseburia intestinalis*, whereas *Clostridium butyricum* made the greatest contribution to butyrate across cohorts and outcome groups (**Figures 3C, D**). In the ANCOM-BC2 analysis of the prespecified butyrate biosynthesis pathways, after adjustment for age, sex, and BMI, acetyl-CoA pathway potential was reduced in the resistant group in both the discovery and external validation cohorts (both *q* < 0.01); the glutarate pathway was reduced only in the discovery cohort (*q* = 0.014), whereas no significant differences were observed in the 4-aminobutyrate or lysine pathways (**Figure 3E**).

**Figure 3:**
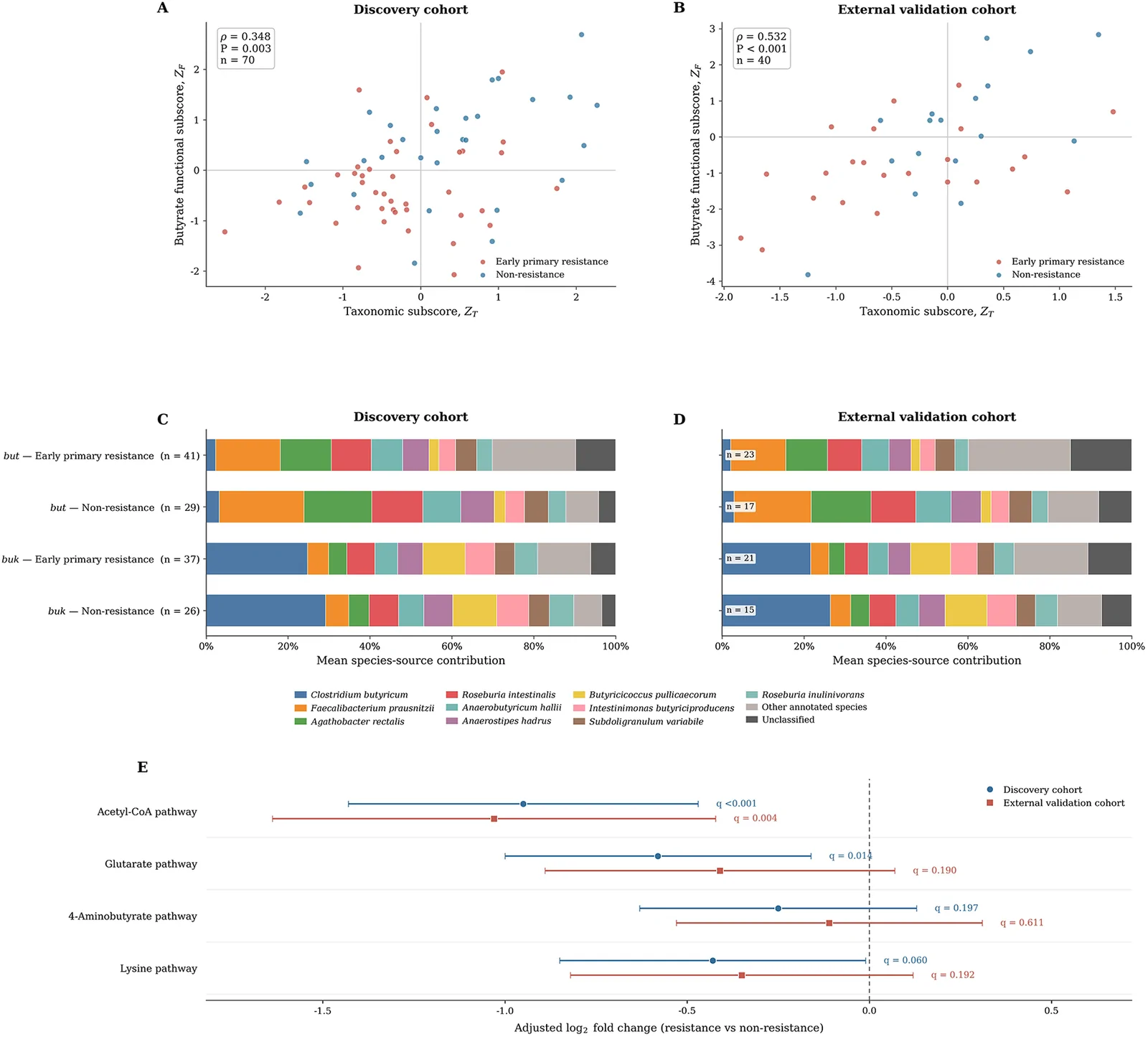
PBAS-related taxonomic–functional consistency and prespecified butyrate biosynthesis pathway potential in the two cohorts. **(A, B)** Spearman correlations between the classical probiotic-associated taxonomic subscore ZT and the butyrate functional subscore ZF. **(C, D)** Species-source composition of *but*/*buk* genes; the 10 species with the highest combined mean contributions in the discovery cohort are shown, and the same list was applied to the external validation cohort. Contribution proportions were calculated within patients and then averaged by outcome group; patients with zero total *buk* TPM were excluded, *but* was detected in all patients, and *A. muciniphila*-derived genes were excluded. **(C, D)** Descriptive only. **(E)** Adjusted log_2_FC estimates and 95% CIs for four prespecified butyrate biosynthesis pathways. ANCOM-BC2 models were adjusted for age, sex, and BMI, and *p*-values were corrected within each cohort using the BH procedure; 95% CIs were unadjusted, and significance was defined as *q* < 0.05. Pathway analysis was used only for supportive functional interpretation and did not contribute to PBAS calculation. BH, Benjamini–Hochberg; BMI, body mass index; CI, confidence interval; log_2_FC, log_2_ fold change; *but*, butyryl-CoA:acetate CoA-transferase gene; *buk*, butyrate kinase gene; PBAS, probiotic–butyrate axis score; TPM, transcripts per million; ZT, taxonomic subscore; ZF, butyrate functional subscore.

### 3.4 Association of PBAS with 90-day early primary resistance and internal validation

The first logistic regression showed that each 1-SD increase in PBAS was associated with lower odds of early primary resistance (OR = 0.39), and the association remained significant after adjustment for clinical variables (OR = 0.37) (both *p* < 0.01), whereas no significant association was observed for *A. muciniphila* (**Table 4**). After correction based on 2,000 patient-level bootstrap repetitions, the AUC of the PBAS main model was 0.733, the Brier score was 0.211, the calibration intercept was − 0.029, and the calibration slope was 1.100. No improvement in performance was observed after the addition of *A. muciniphila*; the differences between apparent and corrected performance were greater for the clinical model and the clinical + PBAS model (**Figures 4A–C**).

**Table 4:** Firth logistic regression of PBAS and prespecified comparators for 90-day early primary resistance.

Model	Predictor (scale or reference category)	OR (95% CI)	*P*-value
A. Taxonomic subscore model	Classical probiotic-associated taxonomic subscore (ZT) (*per 1 discovery-cohort SD*)	0.52 (0.29–0.85)	0.009
B. Functional subscore model	Butyrate functional subscore (ZF) (*per 1 discovery-cohort SD*)	0.43 (0.23–0.72)	0.001
C. PBAS model	Probiotic-butyrate axis score (PBAS) (*per 1 discovery-cohort SD*)	0.39 (0.20–0.67)	< 0.001
D. *A. muciniphila* model	*A. muciniphila* abundance (*per 1 discovery-cohort SD*)	1.21 (0.76–1.97)	0.423
E. PBAS + *A. muciniphila* model	PBAS (*per 1 discovery-cohort SD*)	0.39 (0.21–0.68)	< 0.001
	*A. muciniphila* abundance (*per 1 discovery-cohort SD*)	1.22 (0.73–2.08)	0.448
F. Clinical model	Age (*per 1 year*)	1.06 (0.99–1.14)	0.111
	Male sex (*reference: female*)	0.96 (0.26–3.61)	0.952
	BMI (*per 1 kg/m^2^)*	0.87 (0.74–1.02)	0.083
	Former or current smoking (*reference: never smoking*)	1.07 (0.31–3.77)	0.913
	Squamous-cell carcinoma (*reference: nonsquamous NSCLC*)	1.58 (0.52–4.78)	0.416
	Anti-PD-1-containing combination therapy (*reference: anti-PD-1 monotherapy*)	1.50 (0.49–4.58)	0.479
G. Clinical + PBAS model	PBAS (*per 1 discovery-cohort SD*)	0.37 (0.19–0.71)	0.003
	Age (*per 1 year*)	1.06 (0.99–1.15)	0.107
	Male sex (*reference: female*)	0.94 (0.23–3.95)	0.936
	BMI (*per 1 kg/m^2^*)	0.84 (0.70–1.01)	0.064
	Former or current smoking (*reference: never smoking*)	0.92 (0.23–3.64)	0.904
	Squamous-cell carcinoma (*reference: nonsquamous NSCLC*)	1.63 (0.48–5.52)	0.433
	Anti-PD-1-containing combination therapy (*reference: anti-PD-1 monotherapy*)	1.52 (0.46–5.02)	0.495

All models included 70 patients, of whom 41 experienced early primary resistance. The outcome was coded as resistance = 1 and nonresistance = 0; therefore, an OR < 1 indicates lower odds of resistance with increasing values. Models A–E used Firth bias-reduced logistic regression, with 95% CIs and *p*-values based on profile penalized likelihood and penalized likelihood-ratio tests, respectively. Models F and G were fitted using Firth logistic regression in each of 20 multiply imputed datasets and pooled according to Rubin’s rules; 95% CIs and *p*-values were based on the pooled Wald statistics.

*BMI*, body mass index; *CI*, confidence interval; *NSCLC*, non-small cell lung cancer; *OR*, odds ratio; *PBAS*, probiotic–butyrate axis score.

**Figure 4:**
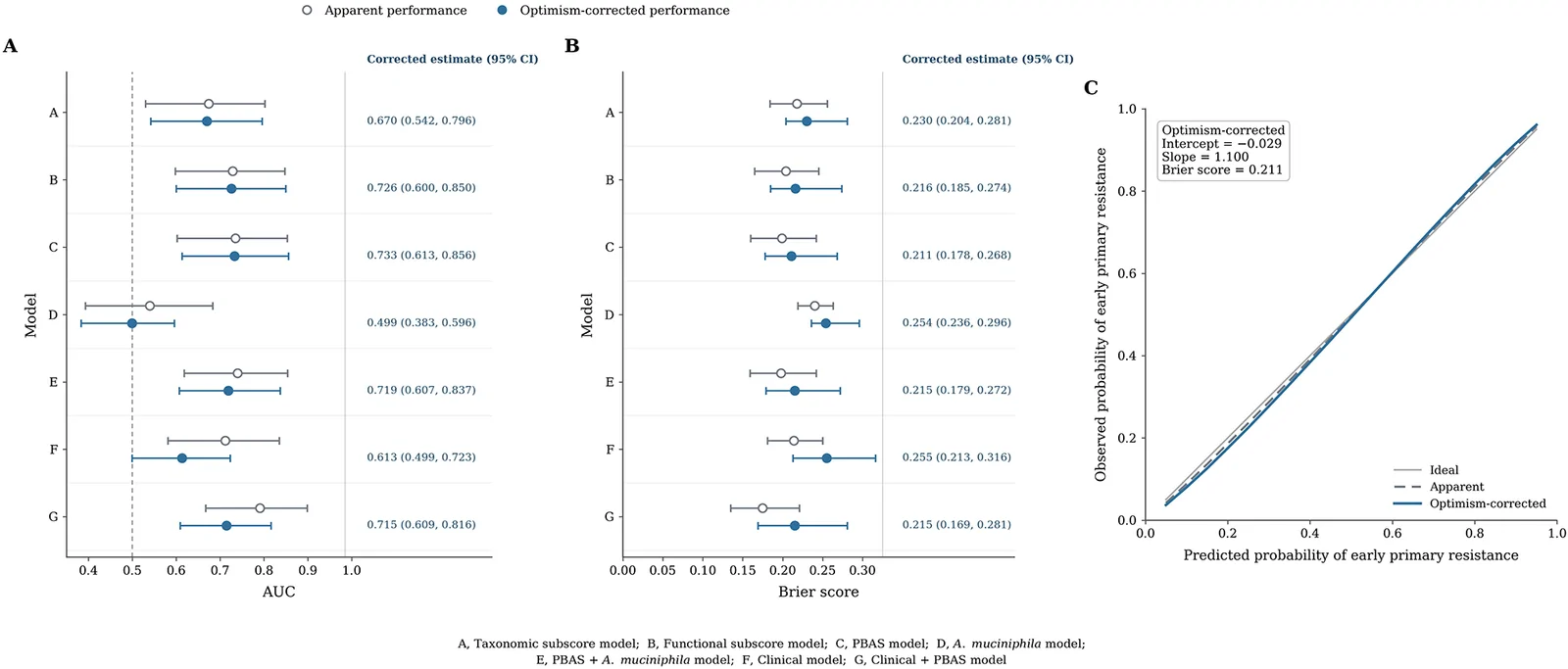
Bootstrap internal validation performance of the PBAS main model and prespecified comparator models in the discovery cohort. **(A)** AUC; **(B)** Brier score. Open circles indicate apparent performance, solid circles indicate optimism-corrected performance based on 2,000 patient-level bootstrap repetitions, and error bars indicate bootstrap percentile 95% CIs. **(C)** Calibration curve of the PBAS main model. Models A–G are defined as follows: A taxonomic subscore model; B functional subscore model; C, PBAS model; D *A. muciniphila* model; E, PBAS + *A. muciniphila* model; F clinical model; and G clinical + PBAS model. AUC, area under the receiver operating characteristic curve; CI, confidence interval; PBAS, probiotic–butyrate axis score.

### 3.5 Independent external validation of the locked PBAS model

The locked PBAS model had an AUC of 0.726 (95% confidence interval [CI], 0.559–0.894), a Brier score of 0.208, an overall calibration bias of − 0.344, and a calibration slope of 0.786 in the external validation cohort. Decision curve analysis showed that the PBAS model had a positive net benefit across threshold probabilities of 0.20–0.75; the Clinical model and Clinical + PBAS model had AUCs of 0.624 (95% CI, 0.440–0.808) and 0.747 (95% CI, 0.586–0.907), respectively, and the addition of PBAS did not yield a statistically significant improvement in discrimination over the Clinical model (ΔAUC = 0.123, DeLong *p* = 0.120) (**Figures 5A–C**). PROBAST+AI assessment showed that the quality concerns for model development and the risk of bias in internal validation of the PBAS main model in the discovery cohort were both low, and applicability concerns were also low. The participants, predictors, and outcome domains of the external validation were at low risk of bias; however, because of the limited validation sample size, the analysis domain and overall risk of bias were rated as high, while applicability concerns remained low (**Supplementary Table S4**).

**Figure 5:**
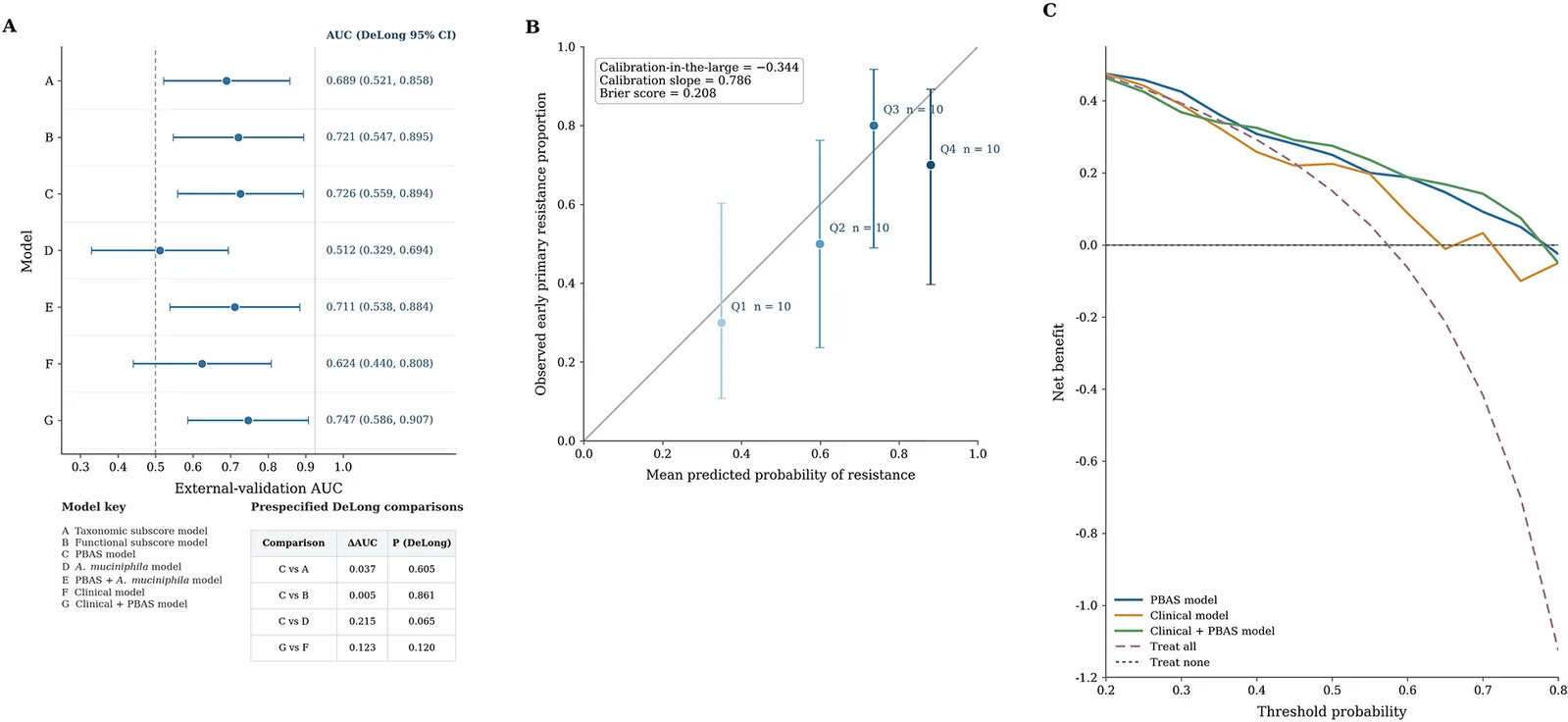
Discrimination, calibration, and clinical net benefit of the locked PBAS model in the independent external validation cohort. The external validation cohort included 40 patients, of whom 23 experienced early primary resistance. **(A)** AUC. **(B)** Calibration of the PBAS model by quartiles of predicted risk; error bars indicate Wilson 95% CIs. **(C)** Decision curves for the PBAS model, clinical model, and clinical + PBAS model across threshold probabilities of 0.20–0.80. All predictions directly used the features, standardization parameters, and model coefficients locked in the discovery cohort, without external restandardization, refitting, or recalibration. Models A–G were the taxonomic subscore model, functional subscore model, PBAS model, *A. muciniphila* model, PBAS + *A. muciniphila* model, clinical model, and clinical + PBAS model, respectively. DCA was used only to evaluate enhanced monitoring or clinical trial screening and not to guide discontinuation of anti-PD-1 therapy. AUC, area under the receiver operating characteristic curve; PBAS, probiotic–butyrate axis score.

### 3.6 Association of PBAS with PFS, robustness, and longitudinal stability

Each 1-SD increase in PBAS was associated with a lower risk of progression or death in both the discovery and external validation cohorts (HR = 0.54 and 0.61, respectively; both *p* < 0.001), and no violation of the proportional hazards assumption was observed (*p*_PH_ = 0.969 and 0.751) (**Figure 6A**). Using the discovery-cohort median PBAS of − 0.18 as the common cutoff, the high-PBAS group had longer PFS in both cohorts (both *p*_log-rank_ < 0.001) (**Figures 6B, C**). The negative association between PBAS and early primary resistance remained directionally consistent when outcomes were defined solely by PFS or response, after further adjustment for *A. muciniphila*, among patients receiving anti-PD-1 monotherapy, and in the complete-case multivariable analysis. Each 1-unit increase in the discovery-cohort SD was also associated with a lower risk of resistance in both cohorts (both *p* < 0.05), whereas no significant association was observed for *buk* (**Table 5**). The longitudinal analysis included 121 fecal samples from 49 patients. Baseline PBAS was lower in the resistant group than in the nonresistant group (*β*_group_ = − 0.863, *p* = 0.001), but the difference in the slopes of PBAS change between the two groups within 30 days was not significant (*β*_interaction_ = − 0.002 SD/day, *p* = 0.495), indicating that no marked change in the between-group difference over time was observed (**Figure 6D**).

**Figure 6:**
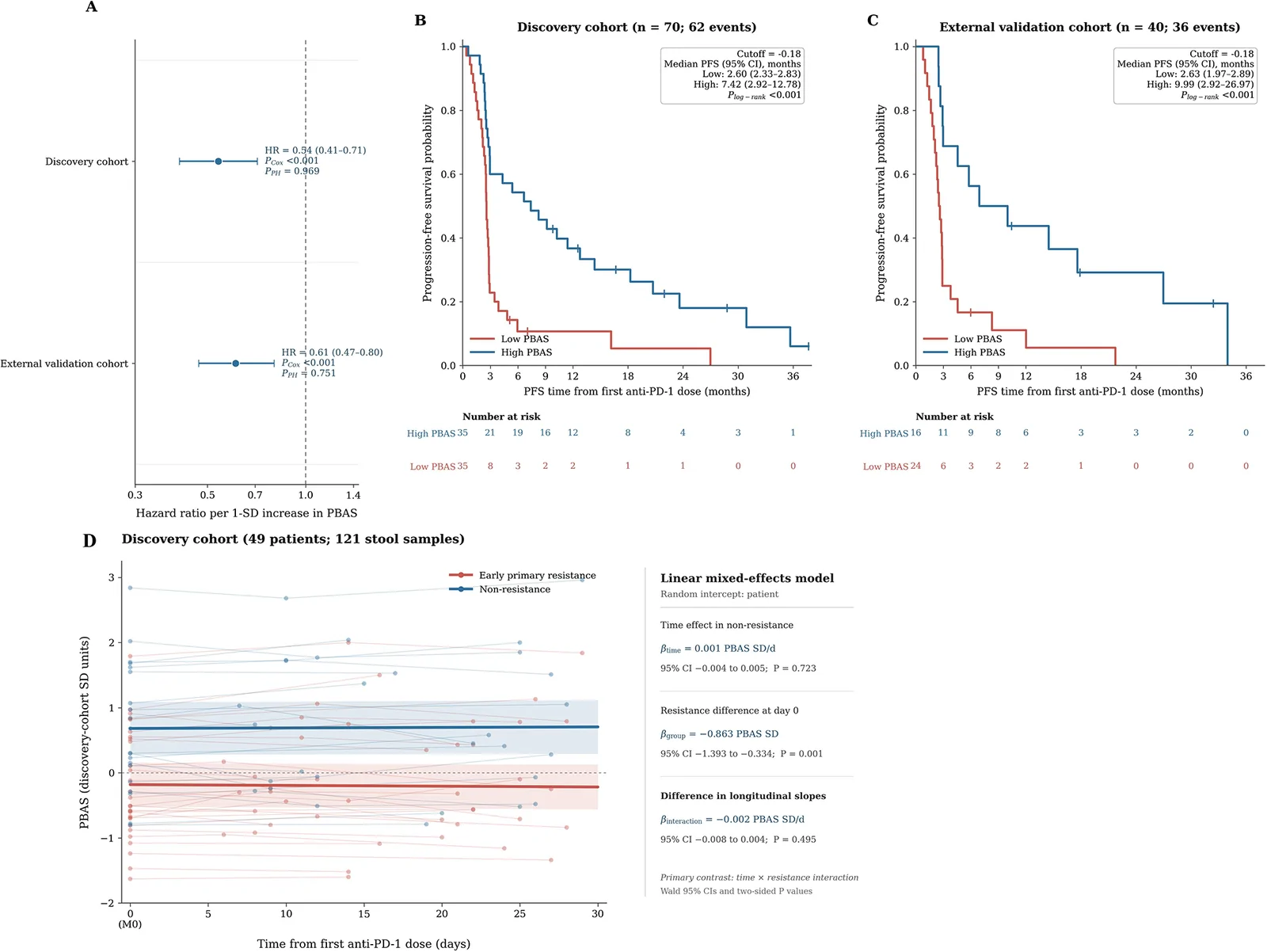
Association of PBAS with progression-free survival and longitudinal changes within 30 days after anti-PD-1 initiation. **(A)** Univariable Cox proportional hazards models; points and error bars indicate HRs and 95% CIs per 1 discovery-cohort SD increase in PBAS. The discovery cohort included 70 patients with 62 PFS events, and the external validation cohort included 40 patients with 36 PFS events. *p*_PH_ was based on the Schoenfeld residual test. **(B, C)** Kaplan–Meier curves for the discovery and external validation cohorts, respectively. Patients were stratified using the discovery-cohort median PBAS of − 0.18 (high PBAS, ≥ 0.18; low PBAS, ≤ − 0.18), and the same cutoff was applied directly to the external cohort. Groups were compared using two-sided log-rank tests, and tick marks indicate censoring. **(D)** Longitudinal PBAS trajectories within 30 days after treatment initiation in the discovery cohort. The analysis included 121 preprogression or predeath stool samples from 49 patients; the most recent pretreatment M0 sample was assigned to day 0. Points and thin lines indicate individual samples and within-patient trajectories, whereas thick lines and shaded bands indicate group mean trajectories and 95% CIs estimated using a random-intercept linear mixed-effects model. The model included time, resistance status, and their interaction; *β*_time_, *β*_group_, and *β*_interaction_ denote the time slope in the nonresistant group, the between-group difference at day 0, and the between-group difference in slopes, respectively. PBAS was calculated using the locked formula and discovery-cohort standardization parameters, and longitudinal samples were not included in the main prediction model. CI, confidence interval; HR, hazard ratio; M0, pretreatment baseline sample; PBAS, probiotic–butyrate axis score; PFS, progression-free survival.

**Table 5:** Prespecified sensitivity analyses of the association between PBAS and 90-day early primary resistance.

Sensitivity analysis	Outcome, patient subset, or model definition	Discovery cohort			External validation cohort		
		*n*/events	OR (95% CI)	*p*-value	*n*/events	OR (95% CI)	*p*-value
PFS-only outcome	Resistance = PFS ≤ 90 days; nonresistance = PFS > 90 days	70/41	0.39 (0.20–0.67)	< 0.001	40/23	0.52 (0.25–0.91)	0.020
Response-only outcome	Resistance = PD or FD; nonresistance = CR, PR, or SD	69/32	0.46 (0.25–0.78)	0.003	39/19	0.54 (0.27–0.93)	0.026
Adjustment for *A. muciniphila*	PBAS effect from a model additionally including *A. muciniphila* abundance	70/41	0.39 (0.21–0.68)	< 0.001	40/23	0.52 (0.25–0.91)	0.021
Anti-PD-1 monotherapy subset	Patients receiving anti-PD-1 monotherapy only	47/26	0.50 (0.24–0.91)	0.023	36/20	0.56 (0.28–0.97)	0.037
Complete-case multivariable analysis	Adjusted for age, sex, BMI, smoking status, histology, and treatment regimen	68/40	0.38 (0.18–0.67)	< 0.001	39/23	0.49 (0.22–0.91)	0.021
*but*-only analysis	Per 1 discovery-cohort SD increase in log_10_(1 + TPM*_but_*)	70/41	0.43 (0.23–0.73)	0.001	40/23	0.61 (0.35–0.96)	0.033
*buk*-only analysis	Per 1 discovery-cohort SD increase in log_10_(1 + TPM*_buk_*)	70/41	0.69 (0.40–1.12)	0.135	40/23	0.66 (0.36–1.12)	0.129

The outcome was coded as resistance = 1. All analyses used Firth bias-reduced logistic regression, with 95% CIs and *p*-values based on profile penalized likelihood and penalized likelihood-ratio tests, respectively. Except for adjustment for *A. muciniphila* and complete-case multivariable analyses, all models included a single predictor. Complete-case models included only patients with complete data for all prespecified clinical covariates and adjusted for age, sex, BMI, smoking status, histology, and treatment regimen. PBAS values from *but*, *buk*, and the external cohort were standardized using discovery-cohort parameters. *p*-values were two sided and nominal, without multiplicity adjustment.

*BMI*, body mass index; *CI*, confidence interval; *OR*, odds ratio; *PBAS*, probiotic–butyrate axis score; *PFS*, progression-free survival; *TPM*, transcripts per million.

## 4 Discussion

Pretreatment classical probiotic-associated taxa and butyrate-producing bacteria were jointly reduced in resistant patients, whereas *E. faecium* was increased, suggesting that early primary resistance does not result from the absence of a single bacterial species but rather reflects a shift of the baseline gut ecological network toward a state of reduced cooperation. *B. longum* and the broad lactobacilli group participate in community metabolism through substrates such as acetate and lactate and are not direct butyrate producers; their reduction may weaken cross-feeding to *F. prausnitzii*, *A. rectalis*, *R. intestinalis*, and *C. butyricum*, thereby impairing substrate supply and the butyrate biosynthesis network (Falony et al., 2006). Such changes may be accompanied by weakened intestinal barrier homeostasis and immune signaling, which is unfavorable for the activation of dendritic cells and effector T cells; however, metagenomic data can support only ecological-functional associations and cannot confirm the direction of causality (Zheng et al., 2020). *E. faecium* is more appropriately regarded as a concomitant marker of ecological dysbiosis, and it cannot be concluded from these findings that it directly mediates resistance. Previous studies have suggested that *Bifidobacterium* enrichment is associated with dendritic cell function, CD8^+^ T-cell priming, and benefit from immune checkpoint blockade, and some NSCLC studies have also observed an association between probiotic use and better efficacy; however, exogenous preparations, strain colonization, and endogenous species abundance do not represent the same exposure (Sivan et al., 2015; Tomita et al., 2020). The absence of a significant difference in *A. muciniphila* in either cohort does not contradict the positive findings from some Western cohorts, because regional diet, background microbiota, antibiotic exclusion, and treatment composition may all contribute to heterogeneity. Excluding it from the score allows the signal to more accurately reflect the complementary value of the classical probiotic-associated ecological network and avoids simplifying efficacy prediction to a single-species biomarker.

Functional alterations in resistant patients were concentrated in the acetyl-CoA pathway and its terminal marker gene, whereas *buk* and the 4-aminobutyrate and lysine pathways did not show cross-cohort consistency. This pattern indicates that the relevant signal represents a selective weakening of the major biosynthetic capacity rather than a general reduction in butyrate metabolism. The taxonomic subscore was positively correlated with the functional subscore. The principal species contributing to the *but* signal were *F. prausnitzii*, *A. rectalis*, and *R. intestinalis*. Together, these findings support the view that classical probiotic-associated ecological features and butyrate biosynthetic potential form a shared functional network. Acetate and lactate produced by *Bifidobacterium* and lactobacilli can be utilized by direct butyrate-producing bacteria, and reductions in these taxa may weaken cross-feeding and reduce substrate supply and metabolic redundancy in the acetyl-CoA pathway (Moens et al., 2017). The *but* pathway is widely distributed in the human gut and may represent the butyrate-forming potential of multiple anaerobic bacteria (Vital et al., 2017). Butyrate production is mainly carried out by specific species and may be less sensitive to an overall decline in community function (Louis et al., 2004). Butyrate can maintain the epithelial barrier, inhibit histone deacetylases, regulate T-cell metabolism, and enhance cytotoxic effects, providing a plausible explanation for its association with anti-PD-1 outcomes (Siddiqui and Cresci, 2021). Previous metagenomic and coculture studies have demonstrated the four major butyrate biosynthesis pathways and acetate–lactate cross-feeding, and experimental models have suggested that butyrate can improve some responses to immune checkpoint blockade (Kang et al., 2023). However, the effects of short-chain fatty acids are influenced by concentration, tissue location, host status, and treatment type, and some CTLA-4 studies have even observed opposite associations (Coutzac et al., 2020). The present findings support an association between lower genetic potential for butyrate biosynthesis and early primary resistance; this cannot be equated with actual butyrate deficiency, nor can it be inferred that butyrate or probiotic supplementation can reverse resistance.

The negative association between PBAS and early primary resistance remained stable after adjustment for clinical variables, under different outcome definitions, in the monotherapy population, in complete-case analyses, and after adjustment for *A. muciniphila*. In practical terms, each 1 discovery-cohort SD increase in PBAS corresponded to approximately 61%–63% lower odds of early primary resistance and 39%–46% lower hazard of progression or death; these estimates represent relative associations rather than absolute risk reductions or causal effects. The association with longer PFS was interpreted as supportive rather than independent evidence because PFS ≤ 90 days contributed to the primary outcome definition. Collectively, these findings suggest that PBAS captures a reproducible pretreatment risk state rather than a chance signal arising from a single analytical setting. No marked between-group difference in slopes was observed in the short-term longitudinal trajectories, further supporting that this score mainly reflects baseline ecological features. PBAS combines classical probiotic-associated taxa with butyrate biosynthetic potential, thereby accounting for both taxonomic specificity and functional redundancy and reducing the loss of transportability caused by species replacement across different regions (Gazzaniga and Kasper, 2025). Its predefined, equally weighted, and low-dimensional construction avoided outcome-driven feature selection and excessive parameter tuning in small samples; therefore, its value lies more in transparency, reproducibility, and direct locked application than in pursuing the highest apparent AUC. Although the external cohort differed in sequencing platform, sequencing depth, BMI, and treatment composition, the model maintained a similar direction of discrimination, indicating preliminary cross-cohort reproducibility of PBAS; however, the calibration bias and effect shrinkage suggested some overestimation of external risk, and the limited sample size also resulted in wide performance intervals; the PROBAST+AI assessment likewise rated the external-validation analysis domain and overall risk of bias as high. Collectively, these limitations make the current evidence insufficient to support definitive clinical validation. Previous microbiome studies of immunotherapy have frequently shown cross-regional inconsistency in species but relative stability in functional features, which is consistent with this ecological-functional integration strategy (Cai et al., 2025). Fecal microbiota transplantation studies suggest that the gut microbiota is modifiable, but this cannot be used to infer that PBAS, probiotics, or butyrate supplementation can reverse resistance in NSCLC (Davar et al., 2021; Baruch et al., 2021). At the present stage, PBAS should be regarded as an exploratory outcome risk marker rather than an established clinical tool and cannot independently determine whether anti-PD-1 therapy should be delayed, discontinued, or withheld.

### 4.1 Limitations

The retrospective secondary analysis of public data limited variable completeness, and only 70 and 40 patients were included in the discovery and external validation cohorts, respectively. Although Firth regression, patient-level bootstrap, and external validation of the locked model reduced overfitting, estimates of external performance remained imprecise, and PROBAST+AI also rated the external validation analysis domain as having a high risk of bias. Diet, dietary fiber, commonly used concomitant medications, treatment line, PD-L1 expression, and tumor mutation burden could not be fully adjusted for, and residual confounding cannot be excluded. Excluding patients with peritreatment antibacterial exposure reduced substantial interference but limited the generalizability of the findings to patients with recent antibacterial use. The two cohorts differed in sequencing platform, read length, sequencing depth, and sample preprocessing procedures, and uniform reanalysis could not completely eliminate technical bias. Lower sequencing depth may preferentially reduce the detection of low-abundance taxa and genes, potentially affecting the taxonomic and functional subscores differently and limiting cross-cohort comparability of absolute *T*, *F*, and PBAS values. The sequencing platform was fully confounded with cohort; therefore, the present data could not separate technical effects from biological cohort differences. Metagenomic data reflect only relative species abundance and gene-encoding potential and cannot confirm strain function, actual butyrate levels, or metabolic flux, nor can they demonstrate that the detected taxa are equivalent to commercial probiotic strains. The absence of metabolomic data, host immune phenotyping, and experimental interventions means that the mechanistic interpretation remains at the level of association. All patients received anti-PD-1 therapy, and no comparator group receiving other treatments was available; therefore, PBAS can be used for outcome risk stratification in this population, but it remains unclear whether it is a general prognostic marker or a treatment-effect marker capable of predicting the relative benefit of anti-PD-1 therapy.

## 5 Conclusion

The combined reduction in pretreatment classical probiotic-associated ecological features and genetic potential for butyrate biosynthesis was associated with a higher risk of early primary resistance to anti-PD-1 therapy in patients with advanced NSCLC. PBAS integrates taxonomic and functional information and showed preliminary cross-cohort discrimination for pretreatment risk stratification. This score should be considered an exploratory risk marker for early primary resistance in patients receiving anti-PD-1 therapy; it cannot yet demonstrate that reduced butyrate potential causes resistance, nor can it serve as a basis for probiotic or butyrate supplementation, treatment discontinuation, or treatment switching. Its clinical translation still requires larger, multicenter prospective validation and studies of metabolic and immune mechanisms.

## Data Availability

The original contributions presented in the study are included in the article/**Supplementary Material**. Further inquiries can be directed to the corresponding author.
