# Supplementary material for: Reduced classical probiotic-associated butyrate biosynthetic potential predicts early primary resistance to anti-PD-1 therapy in advanced non-small cell lung cancer: a shotgun metagenomic study
Source: Front Cell Infect Microbiol. 2026 Oct 28;16:1975315. doi: 10.3389/fcimb.2026.1975315 (PMC13751327; doi:10.3389/fcimb.2026.1975315)
Supplement: Supplementary file 1 [file Table1.docx]

**Supplementary Table S1. Sample-level FASTQ quality control, host-read removal, and metagenomic annotation statistics**

| **Cohort** | **Patient ID** | **Stool sample ID** | **BioSample ID** | **Run ID / merged run list** | **Time point** | **Sampling-to-treatment interval (d)** | **Platform and read structure** | **Raw reads** | **Reads after fastp** | **Q20 (%)** | **Q30 (%)** | **Host reads (%)** | **Non-host reads** | **Kraken2 bacterial classification rate (%)** | **IGC mapping rate (%)** | **Technical QC status** | **Exclusive exclusion reason** |
| --- | --- | --- | --- | --- | --- | --- | --- | --- | --- | --- | --- | --- | --- | --- | --- | --- | --- |
| Discovery cohort | D001 | D-M0-001 | D-BS001 | D-R001 | M0 | -4 | BGISEQ-500, SE50 | 97,152,862 | 93,237,602 | 98.60 | 94.36 | 1.70 | 91,652,563 | 72.90 | 69.20 | Included | — |
| Discovery cohort | D002 | D-M0-002 | D-BS002 | D-R002 | M0 | -7 | BGISEQ-500, SE50 | 123,581,145 | 118,847,987 | 98.21 | 93.54 | 0.73 | 117,980,397 | 88.96 | 85.42 | Included | — |
| Discovery cohort | D003 | D-M0-003 | D-BS003 | D-R003 | M0 | -7 | BGISEQ-500, SE50 | 76,979,935 | 73,839,154 | 97.75 | 92.33 | 1.25 | 72,916,165 | 61.93 | 57.46 | Included | — |
| Discovery cohort | D004 | D-M0-004 | D-BS004 | D-R004 | M0 | -2 | BGISEQ-500, SE50 | 75,071,670 | 70,176,997 | 98.44 | 94.03 | 5.59 | 66,254,103 | 71.74 | 67.99 | Included | — |
| Discovery cohort | D005 | D-M0-005 | D-BS005 | D-R005 | M0 | -2 | BGISEQ-500, SE50 | 68,269,200 | 65,791,028 | 98.66 | 94.49 | 0.25 | 65,626,550 | 72.61 | 68.90 | Included | — |
| Discovery cohort | D006 | D-M0-006 | D-BS006 | D-R006 | M0 | -2 | BGISEQ-500, SE50 | 82,742,079 | 81,203,076 | 99.35 | 96.35 | 3.65 | 78,239,164 | 71.66 | 67.91 | Included | — |
| Discovery cohort | D007 | D-M0-007 | D-BS007 | D-R007 | M0 | -4 | BGISEQ-500, SE50 | 91,422,400 | 88,624,875 | 98.64 | 94.44 | 0.69 | 88,013,363 | 86.65 | 83.15 | Included | — |
| Discovery cohort | D008 | D-M0-008 | D-BS008 | D-R008 | M0 | -2 | BGISEQ-500, SE50 | 58,557,244 | 56,876,651 | 98.26 | 93.65 | 0.50 | 56,592,268 | 88.03 | 84.51 | Included | — |
| Discovery cohort | D009 | D-M0-009 | D-BS009 | D-R009 | M0 | -1 | BGISEQ-500, SE50 | 120,321,505 | 111,802,742 | 96.91 | 90.08 | 2.55 | 108,951,772 | 81.09 | 77.70 | Included | — |
| Discovery cohort | D010 | D-M0-010 | D-BS010 | D-R010 | M0 | -10 | BGISEQ-500, SE50 | 70,269,287 | 65,947,726 | 97.08 | 90.53 | 7.92 | 60,724,666 | 76.38 | 72.83 | Included | — |
| Discovery cohort | D011 | D-M0-011 | D-BS011 | D-R011 | M0 | -3 | BGISEQ-500, SE50 | 118,691,032 | 114,619,930 | 99.13 | 95.67 | 8.31 | 105,095,014 | 87.11 | 83.60 | Included | — |
| Discovery cohort | D012 | D-M0-012 | D-BS012 | D-R012A;D-R012B | M0 | -1 | BGISEQ-500, SE50 | 72,187,469 | 69,364,939 | 97.24 | 90.98 | 1.99 | 67,984,577 | 77.60 | 74.09 | Included | — |
| Discovery cohort | D013 | D-M0-013 | D-BS013 | D-R013 | M0 | -2 | BGISEQ-500, SE50 | 51,272,024 | 49,287,797 | 98.96 | 95.19 | 2.64 | 47,986,599 | 63.14 | 58.77 | Included | — |
| Discovery cohort | D014 | D-M0-014 | D-BS014 | D-R014 | M0 | -1 | BGISEQ-500, SE50 | 83,783,715 | 80,926,690 | 98.24 | 93.59 | 0.56 | 80,473,501 | 82.02 | 78.61 | Included | — |
| Discovery cohort | D015 | D-M0-015 | D-BS015 | D-R015 | M0 | -2 | BGISEQ-500, SE50 | 47,628,555 | 46,061,576 | 98.34 | 93.81 | 0.77 | 45,706,902 | 63.75 | 59.42 | Included | — |
| Discovery cohort | D016 | D-M0-016 | D-BS016 | D-R016 | M0 | -2 | BGISEQ-500, SE50 | 84,744,296 | 80,787,212 | 98.08 | 93.23 | 0.99 | 79,987,419 | 77.31 | 73.79 | Included | — |
| Discovery cohort | D017 | D-M0-017 | D-BS017 | D-R017 | M0 | -10 | BGISEQ-500, SE50 | 113,803,873 | 111,311,568 | 98.37 | 93.87 | 2.36 | 108,684,615 | 87.57 | 84.06 | Included | — |
| Discovery cohort | D018 | D-M0-018 | D-BS018 | D-R018 | M0 | -3 | BGISEQ-500, SE50 | 48,842,577 | 47,362,647 | 98.52 | 94.20 | 7.53 | 43,796,240 | 62.54 | 58.12 | Included | — |
| Discovery cohort | D019 | D-M0-019 | D-BS019 | D-R019 | M0 | -2 | BGISEQ-500, SE50 | 83,700,104 | 82,494,823 | 99.20 | 95.90 | 0.39 | 82,173,093 | 78.74 | 75.27 | Included | — |
| Discovery cohort | D020 | D-M0-020 | D-BS020 | D-R020 | M0 | -5 | BGISEQ-500, SE50 | 90,457,239 | 87,580,699 | 97.16 | 90.75 | 6.36 | 82,010,567 | 75.51 | 71.92 | Included | — |
| Discovery cohort | D021 | D-M0-021 | D-BS021 | D-R021 | M0 | -1 | BGISEQ-500, SE50 | 98,102,040 | 94,079,858 | 98.47 | 94.09 | 0.33 | 93,769,394 | 79.02 | 75.56 | Included | — |
| Discovery cohort | D022 | D-M0-022 | D-BS022 | D-R022 | M0 | -1 | BGISEQ-500, SE50 | 56,128,015 | 53,619,093 | 99.05 | 95.44 | 2.87 | 52,080,225 | 64.36 | 60.07 | Included | — |
| Discovery cohort | D023 | D-M0-023 | D-BS023 | D-R023 | M0 | -3 | BGISEQ-500, SE50 | 92,378,351 | 87,556,201 | 97.66 | 92.10 | 1.29 | 86,426,726 | 83.87 | 80.42 | Included | — |
| Discovery cohort | D024 | D-M0-024 | D-BS024 | D-R024 | M0 | -4 | BGISEQ-500, SE50 | 80,820,694 | 79,454,824 | 98.98 | 95.22 | 0.31 | 79,208,514 | 68.01 | 63.99 | Included | — |
| Discovery cohort | D025 | D-M0-025 | D-BS025 | D-R025 | M0 | -2 | BGISEQ-500, SE50 | 107,289,267 | 103,963,300 | 99.09 | 95.56 | 0.08 | 103,880,129 | 79.30 | 75.85 | Included | — |
| Discovery cohort | D026 | D-M0-026 | D-BS026 | D-R026 | M0 | -2 | BGISEQ-500, SE50 | 115,430,176 | 113,664,094 | 99.28 | 96.12 | 5.98 | 106,866,981 | 84.33 | 80.88 | Included | — |
| Discovery cohort | D027 | D-M0-027 | D-BS027 | D-R027 | M0 | -6 | BGISEQ-500, SE50 | 102,402,146 | 100,415,544 | 99.39 | 96.46 | 1.52 | 98,889,228 | 84.79 | 81.33 | Included | — |
| Discovery cohort | D028 | D-M0-028 | D-BS028 | D-R028 | M0 | -5 | BGISEQ-500, SE50 | 69,308,502 | 67,367,864 | 98.62 | 94.40 | 0.64 | 66,936,710 | 79.87 | 76.44 | Included | — |
| Discovery cohort | D029 | D-M0-029 | D-BS029 | D-R029 | M0 | -5 | BGISEQ-500, SE50 | 125,208,353 | 123,505,519 | 99.51 | 96.80 | 2.83 | 120,010,313 | 85.72 | 82.24 | Included | — |
| Discovery cohort | D030 | D-M0-030 | D-BS030 | D-R030 | M0 | -2 | BGISEQ-500, SE50 | 105,659,640 | 102,542,681 | 98.70 | 94.59 | 1.61 | 100,891,744 | 81.55 | 78.15 | Included | — |
| Discovery cohort | D031 | D-M0-031 | D-BS031 | D-R031 | M0 | 0 | BGISEQ-500, SE50 | 81,778,527 | 77,207,107 | 98.16 | 93.45 | 1.20 | 76,280,622 | 72.03 | 68.29 | Included | — |
| Discovery cohort | D032 | D-M0-032 | D-BS032 | D-R032 | M0 | -1 | BGISEQ-500, SE50 | 93,327,071 | 91,199,214 | 99.54 | 96.91 | 8.69 | 83,274,002 | 66.79 | 62.69 | Included | — |
| Discovery cohort | D033 | D-M0-033 | D-BS033 | D-R033 | M0 | -10 | BGISEQ-500, SE50 | 57,339,508 | 55,797,075 | 98.79 | 94.79 | 3.26 | 53,978,090 | 67.40 | 63.34 | Included | — |
| Discovery cohort | D034 | D-M0-034 | D-BS034 | D-R034 | M0 | -3 | BGISEQ-500, SE50 | 117,056,688 | 109,202,184 | 96.99 | 90.30 | 0.53 | 108,623,412 | 88.50 | 84.97 | Included | — |
| Discovery cohort | D035 | D-M0-035 | D-BS035 | D-R035 | M0 | -1 | BGISEQ-500, SE50 | 89,506,446 | 87,617,860 | 99.62 | 97.14 | 1.42 | 86,373,686 | 72.32 | 68.60 | Included | — |
| Discovery cohort | D036 | D-M0-036 | D-BS036 | D-R036 | M0 | -8 | BGISEQ-500, SE50 | 68,354,494 | 65,923,909 | 98.68 | 94.54 | 0.60 | 65,528,366 | 69.84 | 65.95 | Included | — |
| Discovery cohort | D037 | D-M0-037 | D-BS037 | D-R037 | M0 | -3 | BGISEQ-500, SE50 | 5,314,927 | 4,986,381 | 96.84 | 89.72 | 4.09 | 4,782,643 | 68.41 | 62.57 | Excluded | Non-host reads <5,000,000 |
| Discovery cohort | D038 | D-M0-038 | D-BS038 | D-R038 | M0 | 0 | BGISEQ-500, SE50 | 121,947,231 | 117,886,388 | 97.58 | 91.88 | 9.08 | 107,182,304 | 81.01 | 77.62 | Included | — |
| Discovery cohort | D039 | D-M0-039 | D-BS039 | D-R039 | M0 | 0 | BGISEQ-500, SE50 | 99,143,776 | 92,858,061 | 98.77 | 94.74 | 6.75 | 86,590,142 | 85.26 | 81.79 | Included | — |
| Discovery cohort | D040 | D-M0-040 | D-BS040 | D-R040 | M0 | 0 | BGISEQ-500, SE50 | 54,913,290 | 52,722,250 | 97.83 | 92.55 | 0.94 | 52,226,661 | 74.35 | 70.71 | Included | — |
| Discovery cohort | D041 | D-M0-041 | D-BS041 | D-R041 | M0 | -3 | BGISEQ-500, SE50 | 79,857,502 | 78,436,038 | 98.89 | 95.04 | 0.42 | 78,106,607 | 82.94 | 79.52 | Included | — |
| Discovery cohort | D042 | D-M0-042 | D-BS042 | D-R042 | M0 | -6 | BGISEQ-500, SE50 | 67,062,555 | 64,547,709 | 99.16 | 95.78 | 0.11 | 64,476,707 | 70.44 | 66.60 | Included | — |
| Discovery cohort | D043 | D-M0-043 | D-BS043 | D-R043 | M0 | 0 | BGISEQ-500, SE50 | 86,642,908 | 80,343,969 | 96.74 | 89.63 | 0.90 | 79,620,873 | 76.46 | 72.91 | Included | — |
| Discovery cohort | D044 | D-M0-044 | D-BS044 | D-R044 | M0 | -1 | BGISEQ-500, SE50 | 71,228,727 | 68,180,137 | 98.93 | 95.14 | 1.80 | 66,952,895 | 65.58 | 61.38 | Included | — |
| Discovery cohort | D045 | D-M0-045 | D-BS045 | D-R045 | M0 | -2 | BGISEQ-500, SE50 | 85,687,782 | 82,440,215 | 98.81 | 94.84 | 0.82 | 81,764,205 | 75.22 | 71.62 | Included | — |
| Discovery cohort | D046 | D-M0-046 | D-BS046 | D-R046 | M0 | -1 | BGISEQ-500, SE50 | 52,494,093 | 51,123,997 | 98.85 | 94.94 | 0.14 | 51,052,423 | 61.32 | 56.81 | Included | — |
| Discovery cohort | D047 | D-M0-047 | D-BS047 | D-R047 | M0 | -1 | BGISEQ-500, SE50 | 99,059,314 | 96,553,113 | 98.39 | 93.92 | 1.33 | 95,268,957 | 86.18 | 82.70 | Included | — |
| Discovery cohort | D048 | D-M0-048 | D-BS048 | D-R048 | M0 | -5 | BGISEQ-500, SE50 | 50,058,029 | 49,252,095 | 99.43 | 96.57 | 7.14 | 45,735,495 | 74.06 | 70.41 | Included | — |
| Discovery cohort | D049 | D-M0-049 | D-BS049 | D-R049 | M0 | 0 | BGISEQ-500, SE50 | 64,632,957 | 62,390,193 | 98.50 | 94.14 | 0.22 | 62,252,935 | 82.48 | 79.06 | Included | — |
| Discovery cohort | D050 | D-M0-050 | D-BS050 | D-R050 | M0 | -5 | BGISEQ-500, SE50 | 104,026,291 | 98,793,769 | 98.57 | 94.31 | 4.42 | 94,427,084 | 79.59 | 76.15 | Included | — |
| Discovery cohort | D051 | D-M0-051 | D-BS051 | D-R051 | M0 | 0 | BGISEQ-500, SE50 | 73,153,338 | 70,688,071 | 98.29 | 93.70 | 5.20 | 67,012,291 | 69.23 | 65.30 | Included | — |
| Discovery cohort | D052 | D-M0-052 | D-BS052 | D-R052 | M0 | -10 | BGISEQ-500, SE50 | 87,596,769 | 84,741,114 | 99.01 | 95.33 | 2.74 | 82,419,207 | 74.93 | 71.32 | Included | — |
| Discovery cohort | D053 | D-M0-053 | D-BS053 | D-R053 | M0 | 0 | BGISEQ-500, SE50 | 59,766,026 | 56,203,971 | 97.49 | 91.65 | 1.03 | 55,625,070 | 64.97 | 60.73 | Included | — |
| Discovery cohort | D054 | D-M0-054 | D-BS054 | D-R054A;D-R054B | M0 | -1 | BGISEQ-500, SE50 | 62,202,449 | 60,019,143 | 98.72 | 94.64 | 4.81 | 57,132,222 | 80.73 | 77.33 | Included | — |
| Discovery cohort | D055 | D-M0-055 | D-BS055 | D-R055 | M0 | -4 | BGISEQ-500, SE50 | 74,110,549 | 68,996,921 | 96.82 | 89.85 | 0.28 | 68,803,730 | 73.48 | 69.81 | Included | — |
| Discovery cohort | D056 | D-M0-056 | D-BS056 | D-R056 | M0 | -5 | BGISEQ-500, SE50 | 76,027,251 | 73,639,995 | 98.87 | 94.99 | 9.47 | 66,666,287 | 75.80 | 72.22 | Included | — |
| Discovery cohort | D057 | D-M0-057 | D-BS057 | D-R057 | M0 | -3 | BGISEQ-500, SE50 | 100,770,220 | 98,311,427 | 98.75 | 94.69 | 4.03 | 94,349,476 | 80.44 | 77.03 | Included | — |
| Discovery cohort | D058 | D-M0-058 | D-BS058 | D-R058 | M0 | -8 | BGISEQ-500, SE50 | 94,281,858 | 92,056,806 | 98.55 | 94.25 | 1.12 | 91,025,770 | 76.09 | 72.53 | Included | — |
| Discovery cohort | D059 | D-M0-059 | D-BS059 | D-R059 | M0 | -2 | BGISEQ-500, SE50 | 96,199,484 | 93,323,119 | 98.91 | 95.09 | 2.08 | 91,381,998 | 73.77 | 70.11 | Included | — |
| Discovery cohort | D060 | D-M0-060 | D-BS060 | D-R060 | M0 | -4 | BGISEQ-500, SE50 | 110,547,347 | 107,418,857 | 98.83 | 94.89 | 0.86 | 106,495,055 | 74.64 | 71.02 | Included | — |
| Discovery cohort | D061 | D-M0-061 | D-BS061 | D-R061 | M0 | 0 | BGISEQ-500, SE50 | 77,942,877 | 75,674,739 | 99.24 | 96.01 | 0.45 | 75,334,203 | 78.17 | 74.68 | Included | — |
| Discovery cohort | D062 | D-M0-062 | D-BS062 | D-R062 | M0 | -5 | BGISEQ-500, SE50 | 95,238,297 | 90,095,429 | 97.41 | 91.43 | 0.36 | 89,771,085 | 80.16 | 76.74 | Included | — |
| Discovery cohort | D063 | D-M0-063 | D-BS063 | D-R063 | M0 | -6 | BGISEQ-500, SE50 | 88,561,468 | 84,275,093 | 98.31 | 93.76 | 2.27 | 82,362,048 | 76.74 | 73.20 | Included | — |
| Discovery cohort | D064 | D-M0-064 | D-BS064 | D-R064 | M0 | -1 | BGISEQ-500, SE50 | 78,903,834 | 75,976,502 | 99.32 | 96.24 | 1.89 | 74,540,546 | 66.19 | 62.03 | Included | — |
| Discovery cohort | D065 | D-M0-065 | D-BS065 | D-R065 | M0 | -4 | BGISEQ-500, SE50 | 112,181,275 | 108,153,967 | 98.18 | 93.48 | 0.19 | 107,948,474 | 83.40 | 79.97 | Included | — |
| Discovery cohort | D066 | D-M0-066 | D-BS066 | D-R066 | M0 | 0 | BGISEQ-500, SE50 | 108,922,208 | 105,414,913 | 97.91 | 92.78 | 1.16 | 104,192,100 | 71.05 | 67.26 | Included | — |
| Discovery cohort | D067 | D-M0-067 | D-BS067 | D-R067 | M0 | -1 | BGISEQ-500, SE50 | 65,839,882 | 63,423,558 | 97.33 | 91.20 | 0.16 | 63,322,080 | 78.45 | 74.97 | Included | — |
| Discovery cohort | D068 | D-M0-068 | D-BS068 | D-R068 | M0 | -7 | BGISEQ-500, SE50 | 53,702,931 | 52,209,990 | 98.42 | 93.98 | 1.07 | 51,651,343 | 73.19 | 69.50 | Included | — |
| Discovery cohort | D069 | D-M0-069 | D-BS069 | D-R069 | M0 | -6 | BGISEQ-500, SE50 | 60,987,931 | 57,462,829 | 98.00 | 93.00 | 0.48 | 57,187,007 | 68.62 | 64.65 | Included | — |
| Discovery cohort | D070 | D-M0-070 | D-BS070 | D-R070 | M0 | -9 | BGISEQ-500, SE50 | 126,836,086 | 121,826,061 | 99.47 | 96.69 | 2.17 | 119,182,435 | 77.88 | 74.38 | Included | — |
| Discovery cohort | D071 | D-M0-071 | D-BS071 | D-R071 | M0 | -3 | BGISEQ-500, SE50 | 63,405,521 | 62,118,389 | 99.58 | 97.03 | 2.46 | 60,590,277 | 77.03 | 73.50 | Included | — |
| External validation cohort | E001 | E-T0-001 | E-BS001 | E-R001 | T0 | -11 | Ion Torrent Proton, single-end variable length | 22,831,651 | 21,712,900 | 96.06 | 87.49 | 3.66 | 20,918,208 | 81.01 | 75.02 | Included | — |
| External validation cohort | E002 | E-T0-002 | E-BS002 | E-R002 | T0 | -3 | Ion Torrent Proton, single-end variable length | 20,968,249 | 20,318,233 | 97.53 | 90.87 | 0.63 | 20,190,228 | 88.55 | 83.49 | Included | — |
| External validation cohort | E003 | E-T0-003 | E-BS003 | E-R003 | T0 | 0 | Ion Torrent Proton, single-end variable length | 26,978,567 | 26,298,707 | 98.48 | 93.54 | 1.80 | 25,825,330 | 87.23 | 81.87 | Included | — |
| External validation cohort | E004 | E-T0-004 | E-BS004 | E-R004 | T0 | -28 | Ion Torrent Proton, single-end variable length | 15,278,779 | 13,917,440 | 92.80 | 81.45 | 10.36 | 12,475,593 | 66.29 | 59.74 | Included | — |
| External validation cohort | E005 | E-T0-005 | E-BS005 | E-R005 | T0 | -11 | Ion Torrent Proton, single-end variable length | 16,300,784 | 15,428,692 | 97.24 | 90.14 | 1.05 | 15,266,691 | 77.44 | 70.96 | Included | — |
| External validation cohort | E006 | E-T0-006 | E-BS006 | E-R006 | T0 | -1 | Ion Torrent Proton, single-end variable length | 17,841,768 | 16,769,478 | 93.47 | 82.67 | 0.15 | 16,744,324 | 79.99 | 73.86 | Included | — |
| External validation cohort | E007 | E-T0-007 | E-BS007 | E-R007 | T0 | -8 | Ion Torrent Proton, single-end variable length | 23,513,256 | 22,220,027 | 96.76 | 88.94 | 2.77 | 21,604,532 | 78.46 | 72.12 | Included | — |
| External validation cohort | E008 | E-T0-008 | E-BS008 | E-R008A;E-R008B | T0 | -17 | Ion Torrent Proton, single-end variable length | 22,208,898 | 20,909,677 | 96.95 | 89.42 | 3.88 | 20,098,382 | 84.88 | 79.17 | Included | — |
| External validation cohort | E009 | E-T0-009 | E-BS009 | E-R009 | T0 | -10 | Ion Torrent Proton, single-end variable length | 19,102,862 | 18,195,476 | 97.93 | 91.95 | 6.22 | 17,063,717 | 89.22 | 84.29 | Included | — |
| External validation cohort | E010 | E-T0-010 | E-BS010 | E-R010 | T0 | -16 | Ion Torrent Proton, single-end variable length | 27,550,185 | 25,624,427 | 94.82 | 85.11 | 0.71 | 25,442,494 | 91.87 | 87.53 | Included | — |
| External validation cohort | E011 | E-T0-011 | E-BS011 | E-R011 | T0 | -25 | Ion Torrent Proton, single-end variable length | 18,796,746 | 17,988,486 | 97.04 | 89.66 | 2.10 | 17,610,728 | 82.54 | 76.68 | Included | — |
| External validation cohort | E012 | E-T0-012 | E-BS012 | E-R012 | T0 | -6 | Ion Torrent Proton, single-end variable length | 14,247,933 | 12,855,910 | 93.14 | 82.06 | 8.98 | 11,701,449 | 79.48 | 73.28 | Included | — |
| External validation cohort | E013 | E-T0-013 | E-BS013 | E-R013 | T0 | -2 | Ion Torrent Proton, single-end variable length | 24,661,703 | 23,527,265 | 95.66 | 86.66 | 1.17 | 23,251,996 | 70.98 | 64.43 | Included | — |
| External validation cohort | E014 | E-T0-014 | E-BS014 | E-R014 | T0 | -6 | Ion Torrent Proton, single-end variable length | 19,396,552 | 18,071,767 | 97.14 | 89.90 | 7.60 | 16,698,313 | 73.33 | 66.78 | Included | — |
| External validation cohort | E015 | E-T0-015 | E-BS015 | E-R015 | T0 | -28 | Ion Torrent Proton, single-end variable length | 24,076,652 | 23,282,122 | 98.34 | 93.14 | 8.29 | 21,352,034 | 83.01 | 77.18 | Included | — |
| External validation cohort | E016 | E-T0-016 | E-BS016 | E-R016 | T0 | -3 | Ion Torrent Proton, single-end variable length | 18,199,041 | 17,458,340 | 98.75 | 94.33 | 0.92 | 17,297,723 | 78.97 | 72.70 | Included | — |
| External validation cohort | E017 | E-T0-017 | E-BS017 | E-R017 | T0 | 0 | Ion Torrent Proton, single-end variable length | 19,699,055 | 18,387,098 | 95.50 | 86.33 | 1.68 | 18,078,195 | 86.56 | 81.06 | Included | — |
| External validation cohort | E018 | E-T0-018 | E-BS018 | E-R018 | T0 | -18 | Ion Torrent Proton, single-end variable length | 18,499,514 | 17,010,303 | 96.85 | 89.18 | 9.67 | 15,365,407 | 77.95 | 71.54 | Included | — |
| External validation cohort | E019 | E-T0-019 | E-BS019 | E-R019 | T0 | -9 | Ion Torrent Proton, single-end variable length | 25,819,799 | 24,918,688 | 97.33 | 90.38 | 2.06 | 24,405,363 | 87.89 | 82.68 | Included | — |
| External validation cohort | E020 | E-T0-020 | E-BS020 | E-R020 | T0 | -9 | Ion Torrent Proton, single-end variable length | 15,793,253 | 14,044,940 | 94.48 | 84.50 | 0.31 | 14,001,401 | 67.46 | 60.91 | Included | — |
| External validation cohort | E021 | E-T0-021 | E-BS021 | E-R021 | T0 | -23 | Ion Torrent Proton, single-end variable length | 21,586,745 | 19,943,994 | 96.72 | 88.88 | 0.39 | 19,866,212 | 83.48 | 77.68 | Included | — |
| External validation cohort | E022 | E-T0-022 | E-BS022 | E-R022 | T0 | -11 | Ion Torrent Proton, single-end variable length | 13,217,222 | 12,096,402 | 92.46 | 80.84 | 0.55 | 12,029,872 | 81.60 | 75.68 | Included | — |
| External validation cohort | E023 | E-T0-023 | E-BS023 | E-R023 | T0 | -32 | Ion Torrent Proton, single-end variable length | 20,297,239 | 18,839,897 | 95.79 | 86.94 | 1.93 | 18,476,287 | 72.16 | 65.61 | Included | — |
| External validation cohort | E024 | E-T0-024 | E-BS024 | E-R024 | T0 | -21 | Ion Torrent Proton, single-end variable length | 22,520,690 | 21,910,379 | 98.89 | 94.73 | 1.30 | 21,625,544 | 83.94 | 78.17 | Included | — |
| External validation cohort | E025 | E-T0-025 | E-BS025 | E-R025 | T0 | -16 | Ion Torrent Proton, single-end variable length | 14,758,493 | 13,188,189 | 95.53 | 86.38 | 3.44 | 12,734,515 | 69.81 | 63.26 | Included | — |
| External validation cohort | E026 | E-T0-026 | E-BS026 | E-R026 | T0 | -11 | Ion Torrent Proton, single-end variable length | 5,247,931 | 4,794,768 | 94.86 | 84.73 | 2.56 | 4,671,928 | 78.16 | 69.48 | Excluded | Non-host reads <5,000,000 |
| External validation cohort | E027 | E-T0-027 | E-BS027 | E-R027 | T0 | -4 | Ion Torrent Proton, single-end variable length | 28,131,602 | 27,312,972 | 98.06 | 92.35 | 0.87 | 27,075,349 | 91.21 | 86.72 | Included | — |
| External validation cohort | E028 | E-T0-028 | E-BS028 | E-R028 | T0 | -2 | Ion Torrent Proton, single-end variable length | 25,238,767 | 23,810,253 | 97.43 | 90.62 | 5.54 | 22,491,165 | 90.54 | 85.91 | Included | — |
| External validation cohort | E029 | E-T0-029 | E-BS029 | E-R029 | T0 | -9 | Ion Torrent Proton, single-end variable length | 13,727,964 | 13,141,780 | 95.93 | 87.22 | 0.79 | 13,037,960 | 80.50 | 74.44 | Included | — |
| External validation cohort | E030 | E-T0-030 | E-BS030 | E-R030 | T0 | -4 | Ion Torrent Proton, single-end variable length | 17,899,684 | 16,762,034 | 96.33 | 88.05 | 2.31 | 16,374,831 | 82.07 | 76.18 | Included | — |
| External validation cohort | E031 | E-T0-031 | E-BS031 | E-R031 | T0 | -9 | Ion Torrent Proton, single-end variable length | 20,660,630 | 19,385,869 | 97.65 | 91.16 | 2.55 | 18,891,529 | 74.50 | 67.95 | Included | — |
| External validation cohort | E032 | E-T0-032 | E-BS032 | E-R032 | T0 | -21 | Ion Torrent Proton, single-end variable length | 28,711,627 | 26,845,371 | 96.59 | 88.61 | 0.47 | 26,719,198 | 76.93 | 70.38 | Included | — |
| External validation cohort | E033 | E-T0-033 | E-BS033 | E-R033A;E-R033B | T0 | 0 | Ion Torrent Proton, single-end variable length | 21,898,885 | 21,049,208 | 98.20 | 92.75 | 3.22 | 20,371,424 | 89.88 | 85.10 | Included | — |
| External validation cohort | E034 | E-T0-034 | E-BS034 | E-R034 | T0 | -3 | Ion Torrent Proton, single-end variable length | 21,282,784 | 20,499,578 | 97.79 | 91.55 | 4.85 | 19,505,348 | 85.90 | 80.25 | Included | — |
| External validation cohort | E035 | E-T0-035 | E-BS035 | E-R035 | T0 | -1 | Ion Torrent Proton, single-end variable length | 16,820,863 | 15,249,794 | 94.15 | 83.89 | 1.43 | 15,031,722 | 85.82 | 80.17 | Included | — |
| External validation cohort | E036 | E-T0-036 | E-BS036 | E-R036 | T0 | 0 | Ion Torrent Proton, single-end variable length | 23,143,120 | 21,488,387 | 93.81 | 83.28 | 3.00 | 20,843,735 | 68.64 | 62.09 | Included | — |
| External validation cohort | E037 | E-T0-037 | E-BS037 | E-R037 | T0 | 0 | Ion Torrent Proton, single-end variable length | 26,402,875 | 25,227,947 | 96.46 | 88.33 | 0.23 | 25,169,923 | 85.35 | 79.67 | Included | — |
| External validation cohort | E038 | E-T0-038 | E-BS038 | E-R038 | T0 | -12 | Ion Torrent Proton, single-end variable length | 23,443,398 | 22,111,813 | 96.19 | 87.77 | 4.16 | 21,191,962 | 84.41 | 78.67 | Included | — |
| External validation cohort | E039 | E-T0-039 | E-BS039 | E-R039 | T0 | -13 | Ion Torrent Proton, single-end variable length | 17,331,005 | 16,455,789 | 97.62 | 91.11 | 4.11 | 15,779,456 | 81.52 | 75.60 | Included | — |
| External validation cohort | E040 | E-T0-040 | E-BS040 | E-R040 | T0 | -17 | Ion Torrent Proton, single-end variable length | 19,997,293 | 17,955,569 | 95.16 | 85.72 | 6.91 | 16,714,839 | 75.68 | 69.13 | Included | — |
| External validation cohort | E041 | E-T0-041 | E-BS041 | E-R041 | T0 | -20 | Ion Torrent Proton, single-end variable length | 20,599,824 | 19,528,633 | 98.62 | 93.94 | 1.55 | 19,225,939 | 76.85 | 70.30 | Included | — |

Each row represents one candidate pretreatment stool sample that completed FASTQ download and entered technical quality control. Multiple sequencing runs from the same physical specimen were merged at the FASTQ level and recorded as a run list. Raw reads and reads after fastp were counted as single-end sequence records.. Exclusion reasons were mutually exclusive; when multiple technical issues occurred, the earliest triggered exclusion criterion was recorded. Early primary resistance status was not included. All included samples had ≥5,000,000 non-host reads. IGC, integrated gene catalog; QC, quality control.
