# Supplementary material for: Reduced classical probiotic-associated butyrate biosynthetic potential predicts early primary resistance to anti-PD-1 therapy in advanced non-small cell lung cancer: a shotgun metagenomic study
Source: Front Cell Infect Microbiol. 2026 Oct 28;16:1975315. doi: 10.3389/fcimb.2026.1975315 (PMC13751327; doi:10.3389/fcimb.2026.1975315)
Supplement: Supplementary file 2 [file Table2.docx]

**Supplementary Table S2. Species-level differential abundance analysis in the discovery and external validation cohorts**

| **Cohort** | **NCBI taxon ID** | **Species** | **Resistant** **detection, %** | **Non-resistant** **detection, %** | **Resistant relative abundance,** **median (IQR), %** | **Non-resistant relative abundance,** **median (IQR), %** | **Adjusted** **log₂FC** | **95% CI** | **P** | **BH-adjusted q** |
| --- | --- | --- | --- | --- | --- | --- | --- | --- | --- | --- |
| Discovery cohort | 216816 | *Bifidobacterium longum* | 39.02 | 65.52 | 0.000 (0.000, 0.07) | 0.09 (0.000, 0.23) | -1.620 | -2.286, -0.954 | <0.001 | **<0.001** |
| Discovery cohort | 1492 | *Clostridium butyricum* | 21.95 | 51.72 | 0.000 (0.000, 0.000) | 0.02 (0.000, 0.08) | -1.740 | -2.544, -0.936 | <0.001 | **<0.001** |
| Discovery cohort | 853 | *Faecalibacterium prausnitzii* | 78.05 | 93.10 | 1.89 (0.41, 2.65) | 4.84 (2.64, 5.81) | -1.310 | -1.937, -0.683 | <0.001 | **<0.001** |
| Discovery cohort | 39491 | *Agathobacter rectalis* | 73.17 | 89.66 | 0.87 (0.000, 1.28) | 2.10 (1.43, 2.94) | -1.180 | -1.827, -0.533 | <0.001 | **0.004** |
| Discovery cohort | 1352 | *Enterococcus faecium* | 46.34 | 17.24 | 0.000 (0.000, 0.16) | 0.000 (0.000, 0.000) | 1.520 | 0.638, 2.402 | <0.001 | **0.007** |
| Discovery cohort | 1680 | *Bifidobacterium adolescentis* | 34.15 | 58.62 | 0.000 (0.000, 0.03) | 0.06 (0.000, 0.10) | -1.280 | -2.064, -0.496 | 0.001 | **0.011** |
| Discovery cohort | 166486 | *Roseburia intestinalis* | 48.78 | 72.41 | 0.000 (0.000, 0.28) | 0.57 (0.000, 1.20) | -1.120 | -1.865, -0.375 | 0.003 | **0.021** |
| Discovery cohort | 1308 | *Streptococcus thermophilus* | 21.95 | 44.83 | 0.000 (0.000, 0.000) | 0.000 (0.000, 0.06) | -1.170 | -1.993, -0.347 | 0.005 | **0.031** |
| Discovery cohort | 649756 | *Anaerostipes hadrus* | 63.41 | 82.76 | 0.30 (0.000, 0.90) | 0.73 (0.26, 1.29) | -1.020 | -1.784, -0.256 | 0.009 | **0.046** |
| Discovery cohort | 562 | *Escherichia coli* | 73.17 | 55.17 | 0.37 (0.000, 0.92) | 0.12 (0.000, 0.46) | 1.210 | 0.289, 2.131 | 0.010 | **0.046** |
| Discovery cohort | 573 | *Klebsiella pneumoniae* | 39.02 | 17.24 | 0.000 (0.000, 0.10) | 0.000 (0.000, 0.000) | 1.370 | 0.292, 2.448 | 0.013 | 0.051 |
| Discovery cohort | 208479 | *Enterocloster bolteae* | 48.78 | 27.59 | 0.000 (0.000, 0.17) | 0.000 (0.000, 0.009) | 1.140 | 0.238, 2.042 | 0.013 | 0.051 |
| Discovery cohort | 39488 | *Anaerobutyricum hallii* | 56.10 | 75.86 | 0.09 (0.000, 0.54) | 0.56 (0.23, 1.33) | -0.930 | -1.714, -0.146 | 0.020 | 0.070 |
| Discovery cohort | 33038 | *Mediterraneibacter gnavus* | 68.29 | 48.28 | 0.36 (0.000, 1.06) | 0.000 (0.000, 0.32) | 0.920 | 0.136, 1.704 | 0.021 | 0.070 |
| Discovery cohort | 40518 | *Ruminococcus bromii* | 68.29 | 86.21 | 0.43 (0.000, 0.74) | 1.10 (0.49, 2.40) | -0.910 | -1.714, -0.106 | 0.026 | 0.081 |
| Discovery cohort | 1351 | *Enterococcus faecalis* | 43.90 | 24.14 | 0.000 (0.000, 0.11) | 0.000 (0.000, 0.000) | 1.050 | 0.109, 1.991 | 0.029 | 0.083 |
| Discovery cohort | 214851 | *Subdoligranulum variabile* | 75.61 | 89.66 | 1.04 (0.21, 1.73) | 1.73 (0.85, 4.14) | -0.770 | -1.495, -0.045 | 0.037 | 0.101 |
| Discovery cohort | 84112 | *Eggerthella lenta* | 56.10 | 34.48 | 0.07 (0.000, 0.42) | 0.000 (0.000, 0.08) | 0.880 | 0.037, 1.723 | 0.041 | 0.104 |
| Discovery cohort | 1590 | *Lactiplantibacillus plantarum* | 17.07 | 34.48 | 0.000 (0.000, 0.000) | 0.000 (0.000, 0.02) | -0.930 | -1.832, -0.028 | 0.043 | 0.105 |
| Discovery cohort | 851 | *Fusobacterium nucleatum* | 19.51 | 10.34 | 0.000 (0.000, 0.000) | 0.000 (0.000, 0.000) | 0.980 | -0.017, 1.977 | 0.054 | 0.124 |
| Discovery cohort | 1297617 | *Intestinimonas butyriciproducens* | 31.71 | 48.28 | 0.000 (0.000, 0.03) | 0.000 (0.000, 0.16) | -0.860 | -1.796, 0.076 | 0.072 | 0.157 |
| Discovery cohort | 29466 | *Veillonella parvula* | 43.90 | 27.59 | 0.000 (0.000, 0.22) | 0.000 (0.000, 0.04) | 0.810 | -0.089, 1.709 | 0.077 | 0.162 |
| Discovery cohort | 47715 | *Lacticaseibacillus rhamnosus* | 14.63 | 27.59 | 0.000 (0.000, 0.000) | 0.000 (0.000, 0.02) | -0.760 | -1.694, 0.174 | 0.111 | 0.221 |
| Discovery cohort | 1681 | *Bifidobacterium bifidum* | 17.07 | 31.03 | 0.000 (0.000, 0.000) | 0.000 (0.000, 0.02) | -0.820 | -1.909, 0.269 | 0.140 | 0.268 |
| Discovery cohort | 28025 | *Bifidobacterium animalis* | 12.20 | 24.14 | 0.000 (0.000, 0.000) | 0.000 (0.000, 0.000) | -0.710 | -1.725, 0.305 | 0.170 | 0.300 |
| Discovery cohort | 360807 | *Roseburia inulinivorans* | 39.02 | 55.17 | 0.000 (0.000, 0.09) | 0.13 (0.000, 0.46) | -0.650 | -1.582, 0.282 | 0.172 | 0.300 |
| Discovery cohort | 817 | *Bacteroides fragilis* | 58.54 | 44.83 | 0.12 (0.000, 0.44) | 0.000 (0.000, 0.11) | 0.630 | -0.282, 1.542 | 0.176 | 0.300 |
| Discovery cohort | 35833 | *Bilophila wadsworthia* | 58.54 | 44.83 | 0.11 (0.000, 0.35) | 0.000 (0.000, 0.15) | 0.600 | -0.322, 1.522 | 0.202 | 0.321 |
| Discovery cohort | 357276 | *Phocaeicola dorei* | 68.29 | 58.62 | 0.67 (0.000, 1.38) | 0.31 (0.000, 1.59) | 0.550 | -0.290, 1.390 | 0.200 | 0.321 |
| Discovery cohort | 40520 | *Blautia obeum* | 65.85 | 75.86 | 0.40 (0.000, 0.79) | 0.76 (0.27, 1.53) | -0.500 | -1.311, 0.311 | 0.227 | 0.348 |
| Discovery cohort | 28118 | *Odoribacter splanchnicus* | 39.02 | 48.28 | 0.000 (0.000, 0.18) | 0.000 (0.000, 0.59) | -0.540 | -1.467, 0.387 | 0.253 | 0.376 |
| Discovery cohort | 1582 | *Lacticaseibacillus casei* | 12.20 | 24.14 | 0.000 (0.000, 0.000) | 0.000 (0.000, 0.000) | -0.580 | -1.654, 0.494 | 0.290 | 0.417 |
| Discovery cohort | 1579 | *Lactobacillus acidophilus* | 9.76 | 17.24 | 0.000 (0.000, 0.000) | 0.000 (0.000, 0.000) | -0.440 | -1.502, 0.622 | 0.417 | 0.581 |
| Discovery cohort | 821 | *Phocaeicola vulgatus* | 92.68 | 89.66 | 4.46 (3.02, 7.44) | 3.03 (1.58, 4.62) | 0.280 | -0.479, 1.039 | 0.470 | 0.636 |
| Discovery cohort | 823 | *Parabacteroides distasonis* | 70.73 | 75.86 | 0.76 (0.000, 1.56) | 1.11 (0.13, 1.68) | -0.220 | -0.954, 0.514 | 0.557 | 0.732 |
| Discovery cohort | 165179 | *Prevotella copri* | 39.02 | 44.83 | 0.000 (0.000, 1.81) | 0.000 (0.000, 2.31) | -0.270 | -1.220, 0.680 | 0.578 | 0.738 |
| Discovery cohort | 239935 | *Akkermansia muciniphila* | 60.98 | 55.17 | 0.06 (0.000, 0.28) | 0.04 (0.000, 0.14) | 0.180 | -0.545, 0.905 | 0.627 | 0.779 |
| Discovery cohort | 28116 | *Bacteroides ovatus* | 75.61 | 72.41 | 1.44 (0.40, 3.35) | 1.03 (0.000, 1.76) | 0.180 | -0.600, 0.960 | 0.651 | 0.788 |
| Discovery cohort | 28117 | *Alistipes putredinis* | 60.98 | 65.52 | 0.37 (0.000, 1.56) | 0.53 (0.000, 1.09) | -0.190 | -1.065, 0.685 | 0.670 | 0.791 |
| Discovery cohort | 74426 | *Collinsella aerofaciens* | 75.61 | 72.41 | 0.37 (0.13, 1.32) | 0.68 (0.000, 1.89) | -0.160 | -0.953, 0.633 | 0.693 | 0.794 |
| Discovery cohort | 820 | *Bacteroides uniformis* | 80.49 | 82.76 | 1.57 (0.60, 3.33) | 2.57 (1.70, 5.31) | -0.150 | -1.011, 0.711 | 0.733 | 0.794 |
| Discovery cohort | 46503 | *Parabacteroides merdae* | 58.54 | 62.07 | 0.29 (0.000, 0.82) | 0.19 (0.000, 0.48) | 0.140 | -0.652, 0.932 | 0.729 | 0.794 |
| Discovery cohort | 818 | *Bacteroides thetaiotaomicron* | 82.93 | 79.31 | 2.17 (0.52, 3.08) | 0.96 (0.58, 2.87) | 0.120 | -0.595, 0.835 | 0.742 | 0.794 |
| Discovery cohort | 214856 | *Alistipes finegoldii* | 51.22 | 55.17 | 0.08 (0.000, 0.39) | 0.21 (0.000, 0.57) | -0.120 | -0.916, 0.676 | 0.768 | 0.803 |
| Discovery cohort | 47678 | *Bacteroides caccae* | 43.90 | 48.28 | 0.000 (0.000, 0.18) | 0.000 (0.000, 0.31) | -0.120 | -1.064, 0.824 | 0.803 | 0.804 |
| Discovery cohort | 88431 | *Dorea longicatena* | 60.98 | 65.52 | 0.43 (0.000, 1.41) | 0.37 (0.000, 0.94) | 0.110 | -0.758, 0.978 | 0.804 | 0.804 |
| External validation cohort | 1492 | *Clostridium butyricum* | 17.39 | 47.06 | 0.000 (0.000, 0.000) | 0.000 (0.000, 0.04) | -1.660 | -2.522, -0.798 | <0.001 | **0.003** |
| External validation cohort | 216816 | *Bifidobacterium longum* | 30.43 | 64.71 | 0.000 (0.000, 0.01) | 0.13 (0.000, 0.23) | -1.540 | -2.324, -0.756 | <0.001 | **0.003** |
| External validation cohort | 853 | *Faecalibacterium prausnitzii* | 65.22 | 88.24 | 0.65 (0.000, 1.64) | 3.09 (1.52, 3.67) | -1.290 | -2.035, -0.545 | <0.001 | **0.010** |
| External validation cohort | 1352 | *Enterococcus faecium* | 47.83 | 17.65 | 0.000 (0.000, 0.30) | 0.000 (0.000, 0.000) | 1.580 | 0.639, 2.521 | <0.001 | **0.010** |
| External validation cohort | 39491 | *Agathobacter rectalis* | 65.22 | 82.35 | 0.46 (0.000, 1.11) | 1.39 (0.90, 2.06) | -1.080 | -1.825, -0.335 | 0.004 | **0.038** |
| External validation cohort | 562 | *Escherichia coli* | 73.91 | 52.94 | 0.79 (0.14, 2.14) | 0.06 (0.000, 0.31) | 1.280 | 0.300, 2.260 | 0.010 | 0.063 |
| External validation cohort | 1680 | *Bifidobacterium adolescentis* | 26.09 | 52.94 | 0.000 (0.000, 0.01) | 0.07 (0.000, 0.17) | -1.140 | -2.002, -0.278 | 0.010 | 0.063 |
| External validation cohort | 573 | *Klebsiella pneumoniae* | 39.13 | 17.65 | 0.000 (0.000, 0.18) | 0.000 (0.000, 0.000) | 1.430 | 0.293, 2.567 | 0.014 | 0.064 |
| External validation cohort | 208479 | *Enterocloster bolteae* | 52.17 | 29.41 | 0.06 (0.000, 0.20) | 0.000 (0.000, 0.03) | 1.220 | 0.240, 2.200 | 0.015 | 0.064 |
| External validation cohort | 166486 | *Roseburia intestinalis* | 47.83 | 70.59 | 0.000 (0.000, 0.25) | 0.37 (0.000, 0.78) | -1.020 | -1.843, -0.197 | 0.015 | 0.064 |
| External validation cohort | 1308 | *Streptococcus thermophilus* | 21.74 | 41.18 | 0.000 (0.000, 0.000) | 0.000 (0.000, 0.08) | -1.040 | -1.922, -0.158 | 0.021 | 0.080 |
| External validation cohort | 33038 | *Mediterraneibacter gnavus* | 69.57 | 47.06 | 0.42 (0.000, 1.31) | 0.000 (0.000, 0.27) | 1.010 | 0.128, 1.892 | 0.025 | 0.083 |
| External validation cohort | 649756 | *Anaerostipes hadrus* | 56.52 | 76.47 | 0.14 (0.000, 0.40) | 0.55 (0.32, 1.53) | -0.960 | -1.803, -0.117 | 0.026 | 0.083 |
| External validation cohort | 1351 | *Enterococcus faecalis* | 43.48 | 23.53 | 0.000 (0.000, 0.09) | 0.000 (0.000, 0.000) | 1.100 | 0.100, 2.100 | 0.031 | 0.093 |
| External validation cohort | 39488 | *Anaerobutyricum hallii* | 52.17 | 70.59 | 0.10 (0.000, 0.42) | 0.68 (0.000, 1.18) | -0.910 | -1.753, -0.067 | 0.034 | 0.096 |
| External validation cohort | 84112 | *Eggerthella lenta* | 56.52 | 35.29 | 0.07 (0.000, 0.20) | 0.000 (0.000, 0.06) | 0.920 | 0.018, 1.822 | 0.046 | 0.119 |
| External validation cohort | 40518 | *Ruminococcus bromii* | 65.22 | 82.35 | 0.29 (0.000, 1.03) | 0.61 (0.37, 0.84) | -0.840 | -1.702, 0.022 | 0.056 | 0.139 |
| External validation cohort | 214851 | *Subdoligranulum variabile* | 73.91 | 88.24 | 0.53 (0.10, 1.17) | 1.72 (0.84, 2.88) | -0.730 | -1.534, 0.074 | 0.075 | 0.166 |
| External validation cohort | 357276 | *Phocaeicola dorei* | 69.57 | 58.82 | 0.64 (0.000, 1.07) | 0.20 (0.000, 0.76) | 0.620 | -0.062, 1.302 | 0.075 | 0.166 |
| External validation cohort | 1590 | *Lactiplantibacillus plantarum* | 17.39 | 35.29 | 0.000 (0.000, 0.000) | 0.000 (0.000, 0.03) | -0.840 | -1.800, 0.120 | 0.086 | 0.182 |
| External validation cohort | 817 | *Bacteroides fragilis* | 60.87 | 47.06 | 0.23 (0.000, 0.67) | 0.000 (0.000, 0.38) | 0.700 | -0.126, 1.526 | 0.097 | 0.193 |
| External validation cohort | 29466 | *Veillonella parvula* | 43.48 | 29.41 | 0.000 (0.000, 0.14) | 0.000 (0.000, 0.05) | 0.830 | -0.233, 1.893 | 0.126 | 0.241 |
| External validation cohort | 1297617 | *Intestinimonas butyriciproducens* | 30.43 | 47.06 | 0.000 (0.000, 0.04) | 0.000 (0.000, 0.12) | -0.800 | -1.845, 0.245 | 0.133 | 0.243 |
| External validation cohort | 1681 | *Bifidobacterium bifidum* | 13.04 | 29.41 | 0.000 (0.000, 0.000) | 0.000 (0.000, 0.02) | -0.780 | -1.833, 0.273 | 0.147 | 0.249 |
| External validation cohort | 35833 | *Bilophila wadsworthia* | 60.87 | 47.06 | 0.15 (0.000, 0.22) | 0.000 (0.000, 0.20) | 0.640 | -0.228, 1.508 | 0.148 | 0.249 |
| External validation cohort | 360807 | *Roseburia inulinivorans* | 34.78 | 52.94 | 0.000 (0.000, 0.13) | 0.13 (0.000, 0.45) | -0.680 | -1.703, 0.343 | 0.193 | 0.311 |
| External validation cohort | 28118 | *Odoribacter splanchnicus* | 34.78 | 47.06 | 0.000 (0.000, 0.14) | 0.000 (0.000, 0.34) | -0.580 | -1.527, 0.367 | 0.230 | 0.357 |
| External validation cohort | 47715 | *Lacticaseibacillus rhamnosus* | 13.04 | 23.53 | 0.000 (0.000, 0.000) | 0.000 (0.000, 0.000) | -0.620 | -1.678, 0.438 | 0.251 | 0.368 |
| External validation cohort | 40520 | *Blautia obeum* | 65.22 | 70.59 | 0.48 (0.000, 1.13) | 0.74 (0.000, 1.39) | -0.470 | -1.277, 0.337 | 0.254 | 0.368 |
| External validation cohort | 821 | *Phocaeicola vulgatus* | 91.30 | 88.24 | 4.91 (2.64, 10.69) | 3.82 (1.38, 7.36) | 0.240 | -0.418, 0.898 | 0.475 | 0.665 |
| External validation cohort | 28116 | *Bacteroides ovatus* | 78.26 | 70.59 | 1.23 (0.44, 2.16) | 1.41 (0.000, 1.97) | -0.220 | -1.012, 0.572 | 0.586 | 0.785 |
| External validation cohort | 818 | *Bacteroides thetaiotaomicron* | 86.96 | 82.35 | 4.10 (1.19, 7.14) | 2.14 (0.80, 4.58) | 0.180 | -0.489, 0.849 | 0.598 | 0.785 |
| External validation cohort | 823 | *Parabacteroides distasonis* | 69.57 | 76.47 | 0.68 (0.000, 1.49) | 0.72 (0.30, 0.91) | -0.190 | -1.050, 0.670 | 0.665 | 0.847 |
| External validation cohort | 165179 | *Prevotella copri* | 30.43 | 35.29 | 0.000 (0.000, 0.25) | 0.000 (0.000, 1.09) | -0.180 | -1.215, 0.855 | 0.733 | 0.855 |
| External validation cohort | 28117 | *Alistipes putredinis* | 60.87 | 64.71 | 0.21 (0.000, 1.08) | 0.55 (0.000, 0.98) | -0.150 | -0.962, 0.662 | 0.717 | 0.855 |
| External validation cohort | 820 | *Bacteroides uniformis* | 78.26 | 82.35 | 1.77 (1.41, 4.76) | 1.74 (0.95, 3.74) | 0.140 | -0.627, 0.907 | 0.720 | 0.855 |
| External validation cohort | 214856 | *Alistipes finegoldii* | 47.83 | 52.94 | 0.000 (0.000, 0.67) | 0.10 (0.000, 0.28) | -0.140 | -1.039, 0.759 | 0.760 | 0.863 |
| External validation cohort | 74426 | *Collinsella aerofaciens* | 78.26 | 76.47 | 0.97 (0.38, 1.47) | 0.71 (0.36, 0.95) | 0.110 | -0.734, 0.954 | 0.798 | 0.877 |
| External validation cohort | 46503 | *Parabacteroides merdae* | 56.52 | 58.82 | 0.23 (0.000, 1.05) | 0.68 (0.000, 0.97) | -0.100 | -0.934, 0.734 | 0.814 | 0.877 |
| External validation cohort | 88431 | *Dorea longicatena* | 60.87 | 64.71 | 0.26 (0.000, 1.01) | 0.44 (0.000, 0.99) | -0.090 | -0.984, 0.804 | 0.844 | 0.886 |
| External validation cohort | 47678 | *Bacteroides caccae* | 43.48 | 41.18 | 0.000 (0.000, 0.27) | 0.000 (0.000, 0.23) | 0.050 | -0.935, 1.035 | 0.921 | 0.921 |
| External validation cohort | 239935 | *Akkermansia muciniphila* | 56.52 | 58.82 | 0.03 (0.000, 0.13) | 0.13 (0.000, 0.42) | -0.050 | -0.873, 0.773 | 0.905 | 0.921 |

Relative abundance is expressed as a percentage of Bracken-estimated bacterial reads and is presented as median (IQR) for descriptive purposes only. Effect estimates and P values were obtained from ANCOM-BC2 models adjusted for age, sex, and body mass index. Positive log₂FC indicates higher abundance in the early primary resistance group, whereas negative log₂FC indicates lower abundance. P values were derived from robust Wald tests and adjusted within each cohort using the Benjamini-Hochberg procedure; q<0.05 was considered exploratory significance. All species with an overall prevalence of at least 10% in the corresponding cohort are shown. Rows were ordered by unrounded q values within each cohort and then by absolute log₂FC when q values were identical. Current scientific names and NCBI taxon IDs were retained for traceability. Discovery cohort: resistant, n=41; non-resistant, n=29. External validation cohort: resistant, n=23; non-resistant, n=17. BH, Benjamini-Hochberg; CI, confidence interval; IQR, interquartile range; log₂FC, log₂ fold change.
