# Supplementary material for: Reduced classical probiotic-associated butyrate biosynthetic potential predicts early primary resistance to anti-PD-1 therapy in advanced non-small cell lung cancer: a shotgun metagenomic study
Source: Front Cell Infect Microbiol. 2026 Oct 28;16:1975315. doi: 10.3389/fcimb.2026.1975315 (PMC13751327; doi:10.3389/fcimb.2026.1975315)
Supplement: Supplementary file 3 [file Table3.docx]

**Supplementary Table S3. ANCOM-BC2 differential-abundance analysis of prespecified butyrate-related functional units in the discovery and external validation cohorts**

| **Cohort** | **Functional unit ID** | **Functional gene** | **Functional category** | **Butyrate pathway** | **Detection, resistant (%)** | **Detection, non-resistant (%)** | **Resistant abundance, median (Q1, Q3), TPM** | **Non-resistant abundance, median (Q1, Q3), TPM** | **Adjusted log2FC** | **95% CI** | **P** | **BH q** |
| --- | --- | --- | --- | --- | --- | --- | --- | --- | --- | --- | --- | --- |
| **Discovery cohort** | BUT-HMM / EC 2.8.3.8 | butyryl-CoA:acetate CoA-transferase (but) | but | Shared terminal reaction | 100.00 | 100.00 | 217.78 (153.88, 362.08) | 561.34 (280.84, 999.00) | -1.450 | (-2.253, -0.647) | <0.001 | 0.008 |
| **Discovery cohort** | K00929 / EC 2.7.2.7 | butyrate kinase (buk) | buk | Shared terminal reaction | 90.24 | 89.66 | 18.95 (10.48, 36.15) | 40.69 (15.22, 68.18) | -0.720 | (-1.482, 0.042) | 0.064 | 0.116 |
| **Discovery cohort** | K00626 / EC 2.3.1.9 | acetyl-CoA C-acetyltransferase (thl) | Acetyl-CoA pathway | Acetyl-CoA | 100.00 | 100.00 | 846.32 (613.45, 1138.27) | 1274.18 (941.72, 1616.54) | -0.660 | (-1.131, -0.189) | 0.006 | 0.030 |
| **Discovery cohort** | K00074 / EC 1.1.1.157 | 3-hydroxybutyryl-CoA dehydrogenase (hbd) | Acetyl-CoA pathway | Acetyl-CoA | 97.56 | 100.00 | 287.64 (174.29, 405.77) | 614.83 (388.52, 874.19) | -1.080 | (-1.734, -0.426) | 0.001 | 0.012 |
| **Discovery cohort** | K01692 / EC 4.2.1.17 | enoyl-CoA hydratase/crotonase (cro) | Acetyl-CoA pathway | Acetyl-CoA | 95.12 | 100.00 | 218.91 (126.44, 337.82) | 459.27 (285.16, 687.34) | -1.010 | (-1.654, -0.366) | 0.002 | 0.014 |
| **Discovery cohort** | K01039 / EC 2.8.3.12 | glutaconate CoA-transferase subunit A (gctA) | Glutarate pathway | Glutarate | 82.93 | 93.10 | 38.27 (11.64, 72.91) | 67.84 (31.59, 105.47) | -0.740 | (-1.317, -0.163) | 0.012 | 0.048 |
| **Discovery cohort** | K01040 / EC 2.8.3.12 | glutaconate CoA-transferase subunit B (gctB) | Glutarate pathway | Glutarate | 80.49 | 89.66 | 32.14 (8.27, 60.52) | 58.73 (24.38, 94.16) | -0.690 | (-1.267, -0.113) | 0.019 | 0.063 |
| **Discovery cohort** | K20903 / EC 4.2.1.167 | (R)-2-hydroxyglutaryl-CoA dehydratase subunit A (hgdA) | Glutarate pathway | Glutarate | 73.17 | 82.76 | 17.88 (0.000, 38.64) | 30.91 (8.45, 57.38) | -0.570 | (-1.117, -0.023) | 0.041 | 0.091 |
| **Discovery cohort** | K20904 / EC 4.2.1.167 | (R)-2-hydroxyglutaryl-CoA dehydratase subunit B (hgdB) | Glutarate pathway | Glutarate | 70.73 | 79.31 | 15.41 (0.000, 33.27) | 27.36 (5.81, 49.73) | -0.530 | (-1.076, 0.016) | 0.057 | 0.114 |
| **Discovery cohort** | K01615 / EC 7.2.4.5 | glutaconyl-CoA decarboxylase subunit A (gcdA) | Glutarate pathway | Glutarate | 87.80 | 93.10 | 29.45 (12.87, 55.64) | 43.82 (22.16, 76.19) | -0.410 | (-0.874, 0.054) | 0.083 | 0.126 |
| **Discovery cohort** | K20509 / EC 7.2.4.1 | glutaconyl-CoA decarboxylase subunit B (gcdB) | Glutarate pathway | Glutarate | 85.37 | 89.66 | 23.62 (9.43, 44.78) | 34.91 (15.72, 58.46) | -0.370 | (-0.831, 0.091) | 0.116 | 0.145 |
| **Discovery cohort** | K18120 / EC 1.1.1.61 | 4-hydroxybutyrate dehydrogenase (4hbD) | 4-aminobutyrate pathway | 4-Aminobutyrate | 65.85 | 72.41 | 10.84 (0.000, 25.39) | 15.76 (0.000, 31.48) | -0.290 | (-0.718, 0.138) | 0.184 | 0.204 |
| **Discovery cohort** | K18122 / EC 2.8.3.- | 4-hydroxybutyrate CoA-transferase (4hbt) | 4-aminobutyrate pathway | 4-Aminobutyrate | 58.54 | 62.07 | 6.72 (0.000, 17.84) | 9.18 (0.000, 21.67) | -0.250 | (-0.665, 0.165) | 0.238 | 0.238 |
| **Discovery cohort** | K14534 / EC 4.2.1.120 | 4-hydroxybutyryl-CoA dehydratase (abfH) | 4-aminobutyrate pathway | 4-Aminobutyrate | 68.29 | 75.86 | 9.57 (0.000, 22.14) | 14.36 (2.11, 29.42) | -0.390 | (-0.851, 0.071) | 0.097 | 0.129 |
| **Discovery cohort** | K01843 / EC 5.4.3.2 | lysine 2,3-aminomutase (kamA) | Lysine pathway | Lysine | 78.05 | 86.21 | 25.44 (7.89, 48.76) | 41.83 (19.62, 70.25) | -0.550 | (-1.041, -0.059) | 0.028 | 0.080 |
| **Discovery cohort** | K01844 / EC 5.4.3.3 | beta-lysine 5,6-aminomutase subunit A (kamD) | Lysine pathway | Lysine | 70.73 | 79.31 | 14.72 (0.000, 30.58) | 24.96 (6.34, 45.17) | -0.440 | (-0.923, 0.043) | 0.074 | 0.123 |
| **Discovery cohort** | K18011 / EC 5.4.3.3 | beta-lysine 5,6-aminomutase subunit B (kamE) | Lysine pathway | Lysine | 68.29 | 75.86 | 13.26 (0.000, 27.93) | 22.18 (2.04, 40.75) | -0.410 | (-0.881, 0.061) | 0.088 | 0.126 |
| **Discovery cohort** | K18012 / EC 1.4.1.11 | L-erythro-3,5-diaminohexanoate dehydrogenase (kdd) | Lysine pathway | Lysine | 63.41 | 72.41 | 8.16 (0.000, 19.47) | 15.92 (0.000, 30.18) | -0.590 | (-1.132, -0.048) | 0.033 | 0.083 |
| **Discovery cohort** | K18013 / EC 2.3.1.247 | 3-keto-5-aminohexanoate cleavage enzyme (kce) | Lysine pathway | Lysine | 56.10 | 62.07 | 2.31 (0.000, 10.83) | 5.17 (0.000, 14.92) | -0.330 | (-0.783, 0.123) | 0.153 | 0.180 |
| **Discovery cohort** | K18014 / EC 4.3.1.14 | 3-aminobutyryl-CoA ammonia-lyase (kal) | Lysine pathway | Lysine | 48.78 | 55.17 | 0.000 (0.000, 5.78) | 1.86 (0.000, 7.94) | -0.270 | (-0.693, 0.153) | 0.211 | 0.222 |
| **External validation cohort** | BUT-HMM / EC 2.8.3.8 | butyryl-CoA:acetate CoA-transferase (but) | but | Shared terminal reaction | 100.00 | 100.00 | 137.04 (80.28, 294.12) | 445.68 (185.21, 999.00) | -1.520 | (-2.555, -0.485) | 0.004 | 0.027 |
| **External validation cohort** | K00929 / EC 2.7.2.7 | butyrate kinase (buk) | buk | Shared terminal reaction | 91.30 | 88.24 | 14.14 (2.89, 22.99) | 33.67 (8.55, 65.07) | -0.800 | (-1.919, 0.319) | 0.161 | 0.328 |
| **External validation cohort** | K00626 / EC 2.3.1.9 | acetyl-CoA C-acetyltransferase (thl) | Acetyl-CoA pathway | Acetyl-CoA | 100.00 | 100.00 | 615.28 (408.17, 846.53) | 936.44 (631.52, 1288.73) | -0.720 | (-1.234, -0.206) | 0.006 | 0.030 |
| **External validation cohort** | K00074 / EC 1.1.1.157 | 3-hydroxybutyryl-CoA dehydrogenase (hbd) | Acetyl-CoA pathway | Acetyl-CoA | 95.65 | 100.00 | 198.36 (112.74, 313.52) | 446.19 (276.88, 682.45) | -1.180 | (-1.883, -0.477) | 0.001 | 0.020 |
| **External validation cohort** | K01692 / EC 4.2.1.17 | enoyl-CoA hydratase/crotonase (cro) | Acetyl-CoA pathway | Acetyl-CoA | 95.65 | 100.00 | 154.72 (83.29, 248.65) | 379.84 (219.11, 548.73) | -1.220 | (-1.994, -0.446) | 0.002 | 0.020 |
| **External validation cohort** | K01039 / EC 2.8.3.12 | glutaconate CoA-transferase subunit A (gctA) | Glutarate pathway | Glutarate | 78.26 | 88.24 | 27.94 (5.63, 53.18) | 44.86 (18.72, 72.55) | -0.580 | (-1.205, 0.045) | 0.069 | 0.230 |
| **External validation cohort** | K01040 / EC 2.8.3.12 | glutaconate CoA-transferase subunit B (gctB) | Glutarate pathway | Glutarate | 73.91 | 88.24 | 22.36 (0.000, 46.27) | 38.71 (15.64, 65.29) | -0.540 | (-1.168, 0.088) | 0.092 | 0.230 |
| **External validation cohort** | K20903 / EC 4.2.1.167 | (R)-2-hydroxyglutaryl-CoA dehydratase subunit A (hgdA) | Glutarate pathway | Glutarate | 65.22 | 76.47 | 12.68 (0.000, 29.74) | 20.47 (3.14, 41.86) | -0.380 | (-0.946, 0.186) | 0.188 | 0.335 |
| **External validation cohort** | K20904 / EC 4.2.1.167 | (R)-2-hydroxyglutaryl-CoA dehydratase subunit B (hgdB) | Glutarate pathway | Glutarate | 60.87 | 70.59 | 9.84 (0.000, 24.13) | 14.68 (0.000, 31.22) | -0.290 | (-0.774, 0.194) | 0.240 | 0.343 |
| **External validation cohort** | K01615 / EC 7.2.4.5 | glutaconyl-CoA decarboxylase subunit A (gcdA) | Glutarate pathway | Glutarate | 82.61 | 88.24 | 23.71 (8.39, 48.54) | 29.84 (13.76, 55.31) | -0.180 | (-0.529, 0.169) | 0.312 | 0.390 |
| **External validation cohort** | K20509 / EC 7.2.4.1 | glutaconyl-CoA decarboxylase subunit B (gcdB) | Glutarate pathway | Glutarate | 82.61 | 76.47 | 24.65 (6.73, 46.82) | 22.14 (5.18, 42.35) | 0.110 | (-0.123, 0.343) | 0.355 | 0.418 |
| **External validation cohort** | K18120 / EC 1.1.1.61 | 4-hydroxybutyrate dehydrogenase (4hbD) | 4-aminobutyrate pathway | 4-Aminobutyrate | 60.87 | 64.71 | 7.84 (0.000, 19.42) | 9.11 (0.000, 21.77) | -0.120 | (-0.425, 0.185) | 0.441 | 0.464 |
| **External validation cohort** | K18122 / EC 2.8.3.- | 4-hydroxybutyrate CoA-transferase (4hbt) | 4-aminobutyrate pathway | 4-Aminobutyrate | 56.52 | 52.94 | 4.82 (0.000, 13.68) | 3.96 (0.000, 11.29) | 0.160 | (-0.200, 0.520) | 0.384 | 0.427 |
| **External validation cohort** | K14534 / EC 4.2.1.120 | 4-hydroxybutyryl-CoA dehydratase (abfH) | 4-aminobutyrate pathway | 4-Aminobutyrate | 60.87 | 70.59 | 6.92 (0.000, 16.84) | 9.73 (0.000, 22.61) | -0.310 | (-0.804, 0.184) | 0.219 | 0.337 |
| **External validation cohort** | K01843 / EC 5.4.3.2 | lysine 2,3-aminomutase (kamA) | Lysine pathway | Lysine | 73.91 | 82.35 | 18.37 (0.000, 37.64) | 31.42 (8.26, 56.18) | -0.490 | (-1.026, 0.046) | 0.073 | 0.230 |
| **External validation cohort** | K01844 / EC 5.4.3.3 | beta-lysine 5,6-aminomutase subunit A (kamD) | Lysine pathway | Lysine | 65.22 | 70.59 | 10.24 (0.000, 23.75) | 14.96 (0.000, 29.64) | -0.340 | (-0.819, 0.139) | 0.164 | 0.328 |
| **External validation cohort** | K18011 / EC 5.4.3.3 | beta-lysine 5,6-aminomutase subunit B (kamE) | Lysine pathway | Lysine | 60.87 | 70.59 | 8.71 (0.000, 20.18) | 12.86 (0.000, 26.71) | -0.280 | (-0.709, 0.149) | 0.201 | 0.335 |
| **External validation cohort** | K18012 / EC 1.4.1.11 | L-erythro-3,5-diaminohexanoate dehydrogenase (kdd) | Lysine pathway | Lysine | 56.52 | 64.71 | 4.36 (0.000, 12.54) | 8.77 (0.000, 18.42) | -0.510 | (-1.083, 0.063) | 0.081 | 0.230 |
| **External validation cohort** | K18013 / EC 2.3.1.247 | 3-keto-5-aminohexanoate cleavage enzyme (kce) | Lysine pathway | Lysine | 52.17 | 47.06 | 1.64 (0.000, 7.11) | 0.000 (0.000, 5.26) | 0.140 | (-0.121, 0.401) | 0.293 | 0.390 |
| **External validation cohort** | K18014 / EC 4.3.1.14 | 3-aminobutyryl-CoA ammonia-lyase (kal) | Lysine pathway | Lysine | 39.13 | 47.06 | 0.000 (0.000, 3.92) | 0.000 (0.000, 5.14) | -0.190 | (-0.704, 0.324) | 0.469 | 0.469 |

Abundances are presented as median (Q1, Q3) TPM for descriptive purposes. Effect estimates and P values were obtained from ANCOM-BC2 models adjusted for age, sex, and body mass index. P values were derived from robust Wald tests, and Benjamini-Hochberg correction was performed separately within each cohort. Positive log2FC values indicate higher abundance in the early primary resistance group; q<0.05 was considered exploratory significance. Functional categories were restricted to but, buk, and the acetyl-CoA, glutarate, 4-aminobutyrate, and lysine pathways. A. muciniphila-derived but or buk genes were removed before construction of the functional matrix. K20509 genes were retained only when assigned to the gcdB branch in the frozen target-gene dictionary. Discovery cohort: resistant n=41 and non-resistant n=29; external validation cohort: resistant n=23 and non-resistant n=17. BH, Benjamini-Hochberg; CI, confidence interval; IQR, interquartile range; log2FC, log2 fold change; TPM, transcripts per million.
