# Supplementary material for: Reduced classical probiotic-associated butyrate biosynthetic potential predicts early primary resistance to anti-PD-1 therapy in advanced non-small cell lung cancer: a shotgun metagenomic study
Source: Front Cell Infect Microbiol. 2026 Oct 28;16:1975315. doi: 10.3389/fcimb.2026.1975315 (PMC13751327; doi:10.3389/fcimb.2026.1975315)
Supplement: Supplementary file 4 [file Table4.docx]

**Supplementary Table S4. PROBAST+AI assessment of risk of bias and applicability for the PBAS main model**

| **Evaluation phase** | **Domain** | **Core assessment items** | **Evidence from this study** | **Quality concern / risk of bias** | **Applicability concern** | **Rationale** |
| --- | --- | --- | --- | --- | --- | --- |
| Discovery-cohort model development and internal validation | Participants and data sources | Appropriate data source and study design; representativeness after inclusion and exclusion. | CNP0000636 provided a cohort of antibiotic-unexposed adults with advanced or recurrent NSCLC receiving anti-PD-1 therapy. All identifiable patients with a pretreatment M0 stool sample were screened. Two patients with indeterminate 90-day outcomes and one sample failing the prespecified technical threshold were excluded before model fitting, leaving 70 patients. | L / L | L | The cohort design was appropriate, eligibility criteria matched the target population, and all exclusions were prespecified, documented, and unrelated to observed predictor–outcome associations. |
| Discovery-cohort model development and internal validation | Predictors | Consistent predictor definition, preprocessing, measurement without outcome knowledge, and availability at the intended prediction time. | PBAS components, taxon identifiers, but/buk gene sets, transformations, equal weighting, and standardisation rules were prespecified and frozen before outcome linkage. All predictors were derived from pretreatment stool metagenomes using one reproducible pipeline and were entered as continuous variables. | L / L | L | Predictor construction was outcome-independent, uniformly implemented, technically reproducible, and available before treatment initiation. |
| Discovery-cohort model development and internal validation | Outcome | Appropriate and consistent outcome definition; outcome ascertainment without predictor information; suitable predictor-to-outcome interval. | The outcome was the prespecified study-defined 90-day early primary resistance endpoint based on PD, FD, PFS ≤90 days, or progression/death within 90 days. Non-resistance required CR/PR/SD and PFS >90 days. The same rule was applied to all patients, and microbiome results were not used to determine the outcome. | L / L | L | Outcome ascertainment was clinically based, temporally appropriate, uniform across participants, and independent of PBAS measurement. |
| Discovery-cohort model development and internal validation | Analysis | Sample size; handling of continuous predictors and missing data; avoidance of class-imbalance correction and data leakage; replication of all development steps during resampling; control of optimism. | The primary model used one continuous predictor in 70 patients with 41 resistance events. Firth logistic regression was used; no categorisation, univariable screening, data splitting, SMOTE, or outcome-driven tuning was performed. Clinical covariate missingness was handled by 20-fold multiple imputation. Two thousand patient-level bootstrap samples repeated standardisation, PBAS calculation, model fitting, and performance estimation. | L / L | NA | The event count was adequate for the one-predictor primary model, all modelling steps were reproduced within bootstrap resampling, and optimism-corrected discrimination, calibration, and Brier score were reported. |
| Discovery-cohort model development and internal validation | Overall judgment | Integrated judgment across all four domains. | All development-quality and internal-validation risk-of-bias domains were rated low; applicability concerns were low in the three applicable domains. | L / L | L | No domain was rated high or unclear. The overall judgment was not generated by numerical scoring. |
| Independent external validation | Participants and data sources | Appropriate independent data source and design; representative application of locked eligibility criteria. | The PRJEB22863 NSCLC subset was independent of model development. RCC cases, patients without a true pretreatment T0 sample, protocol-defined antibiotic exposure, indeterminate 90-day outcomes, and one sample failing the technical threshold were excluded according to the frozen protocol, leaving 40 patients with 23 resistance events. | NA / L | L | The external cohort matched the prespecified target population and setting, and selection was completed before examining locked-model performance. |
| Independent external validation | Predictors | Consistent predictor measurement and preprocessing; predictor availability at intended use; absence of outcome-informed recalculation. | The external cohort was processed using the same frozen taxon dictionary, but/buk gene list, feature definitions, and bioinformatic workflow. Discovery-cohort means, standard deviations, score formula, intercept, and coefficient were applied directly; no external re-standardisation, reweighting, or refitting was performed. | NA / L | L | Predictors were measured before treatment and applied exactly as locked. The different sequencing platform constituted a deliberate transportability test rather than outcome-dependent preprocessing. |
| Independent external validation | Outcome | Consistent, clinically appropriate, predictor-independent outcome definition and assessment. | The same 90-day early primary resistance definition used in development was applied to all external patients. Patients censored before 90 days without an event or lacking sufficient response/PFS information were excluded before validation. Predicted probabilities were not used in outcome classification. | NA / L | L | Outcome definition, timing, and ascertainment matched the intended use and were independent of the locked predictions. |
| Independent external validation | Analysis | Avoidance of apparent-only evaluation; adequate validation sample size; appropriate missing-data handling; locked-model evaluation of discrimination, calibration, and net benefit. | The locked model was evaluated without updating in 40 patients, including 23 events and 17 non-events. AUC with DeLong 95% CI, calibration-in-the-large, calibration slope, Brier score, quartile calibration, and decision-curve net benefit were reported. One missing smoking value affected only clinical comparison models and was handled through the prespecified multiple-imputation procedure. | NA / H | NA | Although evaluation methods were appropriate, the small external sample produced wide uncertainty and sparse calibration groups, making discrimination, calibration, and net-benefit estimates insufficiently precise. |
| Independent external validation | Overall judgment | Integrated judgment across all four domains. | Participants, predictors, and outcome were rated at low risk of bias, whereas analysis was rated high because of limited validation sample size. Applicability concerns were low in all three applicable domains. | NA / H | L | A high rating in the analysis domain resulted in an overall high risk-of-bias judgment for external performance estimates; the model remained applicable to the prespecified target population. |

Assessment followed PROBAST+AI. For the discovery phase, the first code denotes concern about model-development quality and the second denotes risk of bias in internal performance evaluation; for external validation, only the second code applies. Applicability is judged only for participants and data sources, predictors, and outcome; the analysis domain is marked NA. L, low; H, high; U, unclear; NA, not applicable. Overall judgments were derived from domain-level assessments and were not calculated by numerical summation. The assessment describes methodological transparency and does not constitute a model-performance metric.
