# Supplementary material for: Reduced classical probiotic-associated butyrate biosynthetic potential predicts early primary resistance to anti-PD-1 therapy in advanced non-small cell lung cancer: a shotgun metagenomic study
Source: Front Cell Infect Microbiol. 2026 Oct 28;16:1975315. doi: 10.3389/fcimb.2026.1975315 (PMC13751327; doi:10.3389/fcimb.2026.1975315)
Supplement: Supplementary file 5 [file Table5.docx]

**Completed STORMS Reporting Checklist**

| **Section/topic** | **Item** | **Reporting recommendation** | **Study-specific reporting and manuscript location** | **Status** |
| --- | --- | --- | --- | --- |
| Abstract | 1.0 | Summarize the rationale, objective, methods, principal results, and appropriately qualified conclusions. | Abstract: Background, Objective, Methods, Results, and Conclusion; the conclusion identifies PBAS as an exploratory outcome-risk marker. | Reported |
| Abstract | 1.1 | State the study design. | Abstract, Methods: dual-cohort, patient-level secondary analysis with a discovery cohort and an independent external-validation cohort. | Reported |
| Abstract | 1.2 | Identify the microbiome measurement or sequencing approach. | Abstract, Methods: pretreatment fecal shotgun metagenomic sequencing data were uniformly reanalysed. | Reported |
| Abstract | 1.3 | State the sampled body site and specimen type. | Abstract/Methods: pretreatment faecal (stool) samples representing the gut microbiome. | Reported |
| Introduction | 2.0 | Explain the scientific background, prior evidence, and biological rationale. | Introduction, paragraphs 1-2: anti-PD-1 resistance, gut microbiome evidence, cross-feeding, and butyrate-related immune mechanisms. | Reported |
| Introduction | 2.1 | State the hypothesis or prespecified objectives and identify exploratory aims. | Introduction, final paragraph: construct PBAS, assess association with 90-day early primary resistance, evaluate discrimination, calibration, net benefit, PFS, and short-term stability. | Reported |
| Methods—Participants | 3.0 | Describe the study design in sufficient detail. | Section 2.1: two-cohort, patient-level secondary analysis; discovery/model-development and locked independent external validation. | Reported |
| Methods—Participants | 3.1 | Define the source population and explain participant and sample selection. | Sections 2.1-2.2 and Figure 1: source projects, patient-sample-run mapping, baseline sample selection, deduplication, and analytic unit. | Reported |
| Methods—Participants | 3.2 | Describe demographic, clinical, geographic, lifestyle, environmental, and treatment characteristics relevant to the microbiome. | Sections 2.1-2.2, Table 1, and Limitations: age, sex, BMI, smoking, histology, treatment regimen, antibiotic exposure; diet and concomitant-medication data were unavailable and acknowledged. | Partly reported |
| Methods—Participants | 3.3 | Report the timing of recruitment, specimen collection, treatment, and follow-up, or explain unavailable calendar dates. | Sections 2.1-2.2 and Table 3: pretreatment M0/T0 sampling, sampling-to-treatment intervals, 90-day outcome window, and PFS follow-up; calendar accrual dates were not available in the public datasets. | Partly reported |
| Methods—Participants | 3.4 | State detailed inclusion and exclusion criteria. | Section 2.2: adult advanced/recurrent NSCLC, anti-PD-1-containing therapy, identifiable pretreatment stool and assessable outcome; antibiotic exposure, post-treatment-only samples, indeterminate outcomes, mapping failures, and QC failures excluded. | Reported |
| Methods—Participants | 3.5 | Report antibiotic and other microbiome-modifying exposures and how they were handled. | Section 2.2: peritreatment antibiotic exposure was excluded; the external-cohort window was 2 months before to 1 month after immunotherapy; other unavailable medication data are acknowledged in Limitations. | Reported |
| Methods—Participants | 3.6 | Report participant, specimen, and read flow, including reasons for exclusion at each stage. | Figure 1, Section 3.1, Tables 2-3, and Supplementary Table S1: patient/sample flow, technical exclusions, non-host-read counts, and exclusive exclusion reasons. | Reported |
| Methods—Participants | 3.7 | For longitudinal analyses, report follow-up procedures and timepoint-specific sample sizes. | Sections 2.9 and 3.6/Figure 6D: 121 pre-progression or pre-death samples from 49 patients within 30 days; longitudinal samples were excluded from the primary model. | Reported |
| Methods—Participants | 3.8 | Describe matching variables and procedures when matching is used. | No case-control matching was used. | Not applicable |
| Methods—Participants | 3.9 | Explain how the analytic study size was determined and any power or precision considerations. | Section 2.7: all eligible patients in both public datasets were included and no post hoc power analysis was performed; small-sample imprecision is discussed in Results and Limitations. | Reported |
| Methods—Laboratory | 4.0 | Describe the specimen and laboratory/sequence-generation workflow sufficiently for interpretation and replication. | Sections 2.1-2.5 and cited source studies describe the stool shotgun-metagenomic datasets and the uniform raw-data reanalysis; original wet-laboratory details remain those of the source cohorts. | Partly reported |
| Methods—Laboratory | 4.1 | Describe specimen collection procedures and timing. | Sections 2.1-2.2 and Table 3 report pretreatment M0/T0 stool selection and timing relative to first anti-PD-1 dose; detailed collection procedures are available only from the source studies. | Partly reported |
| Methods—Laboratory | 4.2 | Describe specimen transport or shipping conditions. | Transport metadata were not available in the public datasets; this limitation is covered under differences in sample preprocessing. | Not available |
| Methods—Laboratory | 4.3 | Describe specimen storage conditions and duration. | Storage metadata were not available for uniform re-reporting; original cohort publications are cited and preprocessing heterogeneity is acknowledged. | Not available |
| Methods—Laboratory | 4.4 | Describe nucleic-acid extraction methods. | The study reanalysed existing FASTQ files and did not repeat DNA extraction; extraction methods belong to the original cohort protocols cited in Section 2.1. | Not applicable to reanalysis |
| Methods—Laboratory | 4.5 | Describe host-DNA removal or microbial-DNA enrichment. | Section 2.3: cleaned reads were aligned to GRCh38.p14 with Bowtie2 very-sensitive mode and human-aligned reads were removed; no additional wet-lab enrichment was performed. | Reported |
| Methods—Laboratory | 4.6 | Describe primers, amplification, and target region when applicable. | Not applicable because the study used shotgun metagenomic rather than amplicon sequencing. | Not applicable |
| Methods—Laboratory | 4.7 | Describe positive controls used during laboratory processing. | Positive-control metadata were not available in the public raw-data records used for secondary analysis. | Not available |
| Methods—Laboratory | 4.8 | Describe negative controls or blanks. | Negative-control metadata were not available in the public raw-data records used for secondary analysis. | Not available |
| Methods—Laboratory | 4.9 | Describe contamination detection and mitigation. | Section 2.3 reports read- and sample-level QC and host depletion; wet-laboratory contaminant-control metadata were unavailable. | Partly reported |
| Methods—Laboratory | 4.10 | Report library/sequencing platform, read structure, read length, and platform-specific handling. | Sections 2.3-2.4 and Table 3: BGISEQ-500 SE50 and Ion Proton single-end data; platform-specific Bracken read-length distribution files were used. | Reported |
| Methods—Laboratory | 4.11 | State the sequencing strategy and report sequencing depth. | Shotgun metagenomics; raw, retained, host-depleted, classified, and IGC-mapped read metrics are reported in Table 3 and Supplementary Table S1. | Reported |
| Methods—Laboratory | 4.12 | Explain abundance calculations and the read/count denominators. | Sections 2.4-2.5: species relative abundance used Bracken-estimated bacterial reads as denominator; gene counts were length-corrected and converted to TPM. | Reported |
| Methods—Laboratory | 4.13 | Describe potential batch effects and measures used to avoid or assess them. | Sections 2.1, 2.4, 2.8, and Limitations: cohorts were not pooled, no cross-cohort batch correction was used, databases/workflows were frozen, and platform/depth heterogeneity and limits on absolute-score comparability are discussed. | Reported |
| Methods—Laboratory | 4.14 | Describe metatranscriptomic methods when used. | No metatranscriptomic data were analysed. | Not applicable |
| Methods—Laboratory | 4.15 | Describe metaproteomic methods when used. | No metaproteomic data were analysed. | Not applicable |
| Methods—Laboratory | 4.16 | Describe metabolomic methods when used. | No metabolomic data were available; absence of measured butyrate/metabolic flux is explicitly acknowledged. | Not applicable |
| Methods—Laboratory | 4.17 | Describe any additional laboratory validation or multi-omic assays relevant to interpretation. | No additional laboratory validation or host immune phenotyping was performed; this is stated in Limitations. | Not applicable |
| Methods—Other measurements | 5.0 | Define non-microbiome variables and their data sources or ascertainment methods. | Sections 2.1-2.2 and 2.7-2.9: clinical variables were abstracted from source studies; early resistance, response, PFS, covariates, and treatment regimen are operationally defined. | Reported |
| Methods—Causal considerations | 6.0 | Justify variables included in adjusted analyses and distinguish association from causal inference. | Sections 2.7-2.8: parsimonious microbiome-feature adjustment and prespecified clinical covariates are justified; no mediation analysis was performed and conclusions are explicitly associational. | Reported |
| Methods—Causal considerations | 6.1 | Discuss selection, survival, or conditioning biases relevant to the design. | Sections 2.1-2.2, PROBAST+AI assessment, and Limitations: exclusions, public-data completeness, antibiotic restriction, outcome ascertainment, residual confounding, and external-validation bias are discussed. | Reported |
| Methods—Bioinformatics/statistics | 7.0 | Describe transformations, normalisation, scaling, and rarefaction decisions. | Sections 2.3-2.6: no rarefaction; relative abundance and TPM definitions; log10 transformations; discovery-cohort Z standardisation; equal-weight PBAS formula. | Reported |
| Methods—Bioinformatics/statistics | 7.1 | Report read- and sample-level quality-control and filtering criteria. | Section 2.3 and Supplementary Table S1: fastp thresholds, read length/ambiguity filters, host depletion, ≥5 million non-host reads, and workflow-failure criteria. | Reported |
| Methods—Bioinformatics/statistics | 7.2 | Describe taxonomic, functional, and sequence-analysis pipelines. | Sections 2.4-2.5: Kraken2/Bracken, frozen RefSeq database, NCBI taxon-ID harmonisation, IGC alignment, HMM/EC criteria, pathway definitions, and exclusion of A. muciniphila-derived target genes. | Reported |
| Methods—Bioinformatics/statistics | 7.3 | Describe all statistical analyses. | Sections 2.7-2.9: Wilcoxon, ANCOM-BC2, Spearman, Firth logistic regression, bootstrap, DeLong, calibration, Brier score, decision curves, Cox models, log-rank tests, and mixed-effects models. | Reported |
| Methods—Bioinformatics/statistics | 7.4 | Explain handling of repeated measures, clustering, or multiple samples per participant. | Sections 2.1 and 2.9: one baseline sample per patient for the primary analysis; runs merged at FASTQ level; patient random-intercept model for longitudinal samples. | Reported |
| Methods—Bioinformatics/statistics | 7.5 | Describe covariate adjustment, missing-data handling, and any imputation. | Sections 2.7-2.8: prespecified covariates, parsimonious high-dimensional adjustment, MICE for covariates with ≤20% missingness, 20 imputations, Rubin pooling, and complete-case sensitivity analysis. | Reported |
| Methods—Bioinformatics/statistics | 7.6 | Describe multiplicity control. | Section 2.7: Benjamini-Hochberg false-discovery-rate control within cohort; exploratory significance at q<0.05; nominal tests are identified where applicable. | Reported |
| Methods—Bioinformatics/statistics | 7.7 | Describe prespecified sensitivity, robustness, and secondary analyses. | Section 2.9 and Table 5: PFS-only, response-only, A. muciniphila adjustment, monotherapy restriction, complete cases, and separate but/buk analyses; PFS is identified as supportive. | Reported |
| Methods—Bioinformatics/statistics | 7.8 | State result-selection criteria and thresholds for statistical interpretation. | Sections 2.7-2.9 and figure/table legends: two-sided α=0.05, q<0.05 for exploratory differential analyses, and specified model/performance comparisons. | Reported |
| Methods—Bioinformatics/statistics | 7.9 | Report software, package, database, reference, and version information. | Section 2.10: Snakemake; fastp 0.23.4, Bowtie2 2.5.4, Kraken2 2.1.3, Bracken 2.9, HMMER 3.4, R 4.4.1, named packages, database/reference versions and MD5 records. | Reported |
| Methods—Reproducibility | 8.0 | State access restrictions and how protected or unavailable materials may be accessed. | The analysis used publicly available de-identified data; no participant-level protected data were newly collected. | Reported |
| Methods—Reproducibility | 8.1 | Provide raw-data accession information. | Section 2.1: CNP0000636 (CNGB Sequence Archive) and PRJEB22863 (European Nucleotide Archive). | Reported |
| Methods—Reproducibility | 8.2 | Describe availability of processed data and derived results. | Main Tables 1-5 and Supplementary Tables S1-S4 provide cohort summaries, QC, differential analyses, and PROBAST+AI assessment; a separate public repository for all intermediate files is not stated. | Partly reported |
| Methods—Reproducibility | 8.3 | Describe availability of analytic metadata and feature definitions. | Sections 2.1, 2.5-2.6, and 2.10 report the sample mapping, frozen taxon/gene definitions, parameters, and version-control procedures; public deposition is not stated. | Partly reported |
| Methods—Reproducibility | 8.4 | Describe availability of analysis code and computational workflows. | Section 2.10 states that Snakemake workflows and analysis scripts were version-controlled; a public code repository or access route is not specified. | Partly reported |
| Methods—Reproducibility | 8.5 | Describe protocols, containers, seeds, parameter files, and other materials needed for reproducibility. | Section 2.10 reports container/reference MD5 values, fixed random seeds, frozen code/parameters, and version control; public access to these materials is not specified. | Partly reported |
| Results—Descriptive data | 9.0 | Report descriptive characteristics of the analytic population. | Section 3.1 and Tables 1-3: demographics, BMI, smoking, histology, treatment composition, outcomes, timing, sequencing depth, and QC metrics, including SMDs. | Reported |
| Results—Microbiome outcomes | 10.0 | Provide descriptive microbiome results by relevant groups and timepoints. | Figures 2-3 and Section 3.2-3.3 report PBAS-related taxonomic/functional distributions, detection, correlations, gene sources, and pathway estimates; broad alpha/beta-diversity analyses were outside the prespecified scope. | Partly reported |
| Results—Microbiome outcomes | 10.1 | Use identifiable and standardised taxonomic or functional units. | NCBI taxon IDs, harmonised species names, IGC gene identifiers, KO/EC/HMM definitions, and pathway labels are reported in Methods and Supplementary Tables S2-S3. | Reported |
| Results—Microbiome outcomes | 10.2 | Report the magnitude, direction, and uncertainty of differential-abundance findings. | Section 3.2, Figure 3E, and Supplementary Tables S2-S3 report adjusted log2 fold changes, 95% CIs, P values, and BH q values. | Reported |
| Results—Microbiome outcomes | 10.3 | Report functional or metabolic-potential findings. | Sections 3.2-3.3, Figure 3, and Supplementary Table S3 report but/buk, acetyl-CoA and other pathway signals, and taxonomic sources of terminal genes. | Reported |
| Results—Microbiome outcomes | 10.4 | Report secondary, null, and robustness findings rather than only positive results. | Sections 3.2-3.6, Tables 4-5, and supplementary tables report null A. muciniphila/buk/pathway findings, comparator models, PFS, sensitivity analyses, and longitudinal results. | Reported |
| Discussion | 11.0 | Summarise the principal findings in relation to the objectives. | Discussion, paragraphs 1-3: ecological-functional pattern, PBAS association, external performance, PFS support, and longitudinal stability. | Reported |
| Discussion | 12.0 | Interpret findings in relation to biological mechanisms and previous studies without overstating causality. | Discussion, paragraphs 1-3: cross-feeding, butyrate biology, regional heterogeneity, alternative findings, and explicit separation of genetic potential from measured butyrate or intervention effects. | Reported |
| Discussion | 13.0 | Discuss study and method limitations. | Dedicated Limitations paragraph: retrospective public-data analysis, sample size, residual confounding, antibiotic restriction, platform heterogeneity, missing omics/immune data, and no comparator treatment group. | Reported |
| Discussion | 13.1 | Describe potential biases and how they may influence the findings. | Results/Discussion and Supplementary Table S4: external-validation imprecision, calibration bias/shrinkage, PROBAST+AI high risk in the analysis domain, technical bias, selection, measurement, and residual confounding. | Reported |
| Discussion | 13.2 | Discuss generalisability and transportability. | Discussion and Limitations: cross-platform/cohort differences, restricted antibiotic-unexposed population, exploratory status, limited absolute-score comparability, and need for multicentre validation. | Reported |
| Discussion | 14.0 | Describe priorities for future research. | Conclusion/Discussion: larger multicentre prospective validation, metabolomic and immune-mechanistic studies, and evaluation before clinical use. | Reported |
| Other information | 15.0 | Report study funding and funder role. | Funding statement: no financial support was received. | Reported |
| Other information | 15.1 | Provide acknowledgements where applicable. | No separate acknowledgements are reported. | Not applicable |
| Other information | 15.2 | Declare conflicts of interest. | Conflict-of-interest statement: no known conflicts were declared. | Reported |
| Other information | 16.0 | State where supplementary materials can be accessed. | Supplementary Tables S1-S4 and the completed STORMS and TRIPOD+AI checklists are cited in the manuscript and supplied with the submission. | Reported |
| Other information | 17.0 | State where study data can be accessed. | Raw data are available under CNP0000636 and PRJEB22863; derived results are reported in the main and supplementary tables. | Reported |
