# Supplementary material for: Reduced classical probiotic-associated butyrate biosynthetic potential predicts early primary resistance to anti-PD-1 therapy in advanced non-small cell lung cancer: a shotgun metagenomic study
Source: Front Cell Infect Microbiol. 2026 Oct 28;16:1975315. doi: 10.3389/fcimb.2026.1975315 (PMC13751327; doi:10.3389/fcimb.2026.1975315)
Supplement: Supplementary file 6 [file Table6.docx]

**Completed TRIPOD+AI Reporting Checklist**

D = model development; E = model evaluation. The present study includes model development, internal validation, and independent external evaluation. Items that were not assessed or were not available in the public source datasets are identified explicitly.

| **Section/topic** | **Item** | **D/E** | **Reporting recommendation** | **Study-specific reporting and manuscript location** | **Status** |
| --- | --- | --- | --- | --- | --- |
| Title | 1 | D;E | Identify model development and/or evaluation, the target population, and the outcome. | Title identifies advanced NSCLC, early primary resistance to anti-PD-1 therapy, and a shotgun-metagenomic prediction study; the manuscript includes both model development and independent evaluation. | Reported |
| Abstract | 2 | D;E | Provide an informative structured summary following TRIPOD+AI for Abstracts. | Structured Abstract reports background, objective, dual-cohort design, sample sizes/events, PBAS construction, modelling, internal and external performance, PFS, and an appropriately qualified conclusion. | Reported |
| Introduction—Background | 3a | D;E | Explain the healthcare context, prognostic/diagnostic role, rationale, and relation to existing models. | Introduction paragraphs 1-2 describe early anti-PD-1 resistance, limitations of established biomarkers and single-species microbiome signals, and the rationale for an ecological-functional prognostic risk marker. | Reported |
| Introduction—Background | 3b | D;E | Describe the target population, intended purpose, and intended users within the care pathway. | Advanced/recurrent NSCLC patients receiving anti-PD-1-containing therapy; intended as exploratory pretreatment outcome-risk stratification rather than a treatment-selection tool. | Reported |
| Introduction—Background | 3c | D;E | Describe known health inequalities or relevant sociodemographic differences. | Cross-regional and demographic heterogeneity is acknowledged, but the available sample size did not support formal fairness or health-inequality analyses. | Partly reported |
| Introduction—Objectives | 4 | D;E | Specify whether the study develops, evaluates, or both develops and evaluates a model. | Introduction final paragraph and Section 2.1: PBAS was developed and internally validated in CNP0000636, then evaluated once in the independent PRJEB22863 cohort. | Reported |
| Methods—Data | 5a | D;E | Describe development and evaluation data sources separately, why they were used, and their representativeness. | Section 2.1 describes CNP0000636 as the discovery/model-development cohort and PRJEB22863 as the independent external-validation cohort, with source repositories and cohort-specific selection. | Reported |
| Methods—Data | 5b | D;E | Report dates of participant accrual/data collection and end of follow-up when available. | Relative timing of stool collection, treatment initiation, the 90-day outcome window, and PFS follow-up is reported; calendar accrual dates were not available from the public secondary datasets. | Partly reported |
| Methods—Participants | 6a | D;E | Describe the clinical setting, number of centres, and geographic context. | Public NSCLC cohorts and their source studies are identified; discovery and external cohorts represent different geographic/technical settings. Detailed centre-level information is available from the original studies rather than fully repeated. | Partly reported |
| Methods—Participants | 6b | D;E | State participant eligibility criteria. | Section 2.2 gives detailed inclusion/exclusion criteria, including age, disease status, anti-PD-1 therapy, pretreatment stool, outcome ascertainment, antibiotic exposure, and technical QC. | Reported |
| Methods—Participants | 6c | D;E | Describe treatments received and how treatment was handled in model development/evaluation. | Anti-PD-1 monotherapy versus anti-PD-1-containing combination therapy is reported, included in adjusted models, compared across cohorts, and examined in a monotherapy-only sensitivity analysis. | Reported |
| Methods—Data preparation | 7 | D;E | Describe preprocessing and quality checks, including consistency across relevant demographic groups. | Sections 2.3-2.5 and Supplementary Table S1 detail FASTQ QC, host depletion, taxonomic and IGC processing, thresholds, and frozen databases. No demographic-group-specific preprocessing was used. | Reported |
| Methods—Outcome | 8a | D;E | Define the predicted outcome, time horizon, ascertainment, rationale, and consistency across groups. | Section 2.2 defines study-specific 90-day early primary resistance from response, progression/death, and PFS criteria; the same operational definition was applied across cohorts, with response-only and PFS-only sensitivity analyses. | Reported |
| Methods—Outcome | 8b | D;E | If outcome ascertainment is subjective, describe assessor qualifications and relevant characteristics. | Outcome data were obtained from the original clinical cohorts; assessor-level qualifications and demographics were not available in the public secondary datasets. | Not available |
| Methods—Outcome | 8c | D;E | Describe blinding of outcome assessment or model evaluation. | External outcomes remained blinded until sample selection, feature definitions, scoring formula, model coefficients, and analysis code were frozen. | Reported |
| Methods—Predictors | 9a | D | Explain the initial predictor set and any preselection before model building. | Sections 2.5-2.6: classical probiotic-associated taxa and but/buk were predefined from biological rationale and literature; A. muciniphila was excluded from PBAS and retained as a comparator; no outcome-driven feature selection was used. | Reported |
| Methods—Predictors | 9b | D;E | Define each predictor and how and when it was measured, including blinding where relevant. | Sections 2.3-2.6 define pretreatment measurement, species and gene quantification, transformations, discovery-cohort standardisation, equal weighting, and locked application to the external cohort. | Reported |
| Methods—Predictors | 9c | D;E | If predictor measurement is subjective, describe assessor qualifications and characteristics. | Predictors were generated through automated bioinformatic pipelines with frozen databases and thresholds; subjective human interpretation was not required. | Not applicable |
| Methods—Sample size | 10 | D;E | Explain how development and evaluation sample sizes were obtained and justify precision, including calculations if performed. | All eligible participants in fixed public datasets were included (70 development, 40 evaluation); no post hoc power analysis was performed. Limited development information and external precision are explicitly acknowledged. | Reported |
| Methods—Missing data | 11 | D;E | Describe handling of missing data and reasons for omitted observations. | Section 2.7: outcome, PBAS, and components were not imputed; clinical covariates with ≤20% missingness underwent MICE (20 datasets, Rubin pooling); variables with >20% missingness were omitted; complete-case sensitivity analysis was performed. | Reported |
| Methods—Analysis | 12a | D | Explain how data were assigned to model development and evaluation and whether partitioning was used. | Section 2.1: cohorts were not pooled; CNP0000636 was used for development/internal bootstrap validation and PRJEB22863 solely for one locked external evaluation; no sample splitting was used. | Reported |
| Methods—Analysis | 12b | D | Describe predictor functional form, transformation, scaling, and standardisation. | Section 2.6: log10 transformations, discovery-cohort Z scores, equal-weight PBAS*, and final discovery-cohort standardisation; continuous PBAS was used in primary models. | Reported |
| Methods—Analysis | 12c | D | Specify model type, rationale, model-building steps, tuning, and internal validation. | Section 2.8: Firth bias-reduced logistic regression, one-predictor primary model, prespecified comparator/adjusted models, no LASSO/random forest/oversampling/synthetic cases, and 2,000 patient-level bootstrap repetitions. | Reported |
| Methods—Analysis | 12d | D;E | Describe how heterogeneity across clusters/settings was handled or quantified. | Cohorts were analysed separately; SMDs and technical/clinical differences were reported; the locked model was transferred without batch correction or recalibration. No cluster-specific parameter model was fitted. | Reported |
| Methods—Analysis | 12e | D;E | Specify performance measures, plots, rationale, and model comparisons. | Section 2.8: AUC, calibration intercept/slope/plots, Brier score, decision-curve net benefit, bootstrap optimism correction, external DeLong CIs, and DeLong model comparisons. | Reported |
| Methods—Analysis | 12f | E | Describe any model updating or recalibration after evaluation. | No external updating, refitting, restandardisation, or recalibration was performed. | Reported |
| Methods—Analysis | 12g | E | Explain how predictions were generated in the evaluation dataset. | The external cohort used the locked species dictionary, gene set, discovery means/SDs, PBAS formula, and model coefficients; no external outcomes were used to alter predictions. | Reported |
| Methods—Class imbalance | 13 | D;E | Describe any methods used to address class imbalance and subsequent recalibration. | No oversampling, synthetic cases, or class weighting was used; event frequencies are reported and Firth estimation addressed small-sample bias rather than class imbalance. | Reported |
| Methods—Fairness | 14 | D;E | Describe any model-fairness analyses or mitigation and their rationale. | No formal fairness analysis was performed because subgroup sizes were insufficient; this limits inference across demographic subgroups. | Not assessed |
| Methods—Model output | 15 | D | Specify model output and any classification thresholds. | The primary model generated individual probabilities of 90-day early resistance. PBAS remained continuous; the discovery median was used only for Kaplan-Meier presentation, not as a clinical classification threshold. | Reported |
| Methods—Development versus evaluation | 16 | D;E | Identify differences between development and evaluation data in setting, eligibility, outcome, and predictors. | Sections 3.1, Tables 1-3, Discussion, and Limitations describe differences in platform, depth, BMI, treatment composition, geography/background microbiota, while retaining consistent eligibility, outcome, and locked predictor definitions. | Reported |
| Methods—Ethics | 17 | D;E | Report ethics review and informed-consent arrangements or waivers. | Section 2.11: only publicly available de-identified data were used, with no new recruitment, sampling, or intervention; renewed ethics review and consent could be waived. | Reported |
| Open science—Funding | 18a | D;E | State funding sources and funder roles. | Funding: no financial support was received. | Reported |
| Open science—Conflicts | 18b | D;E | Declare conflicts of interest and financial disclosures. | Conflict of interest: no known conflicts were declared. | Reported |
| Open science—Protocol | 18c | D;E | Provide protocol access or state that no separate protocol was prepared. | No standalone protocol was prepared; prespecified feature definitions, analysis rules, model locking, and reproducibility procedures are described in Sections 2.1, 2.5-2.10. | Reported in checklist |
| Open science—Registration | 18d | D;E | Provide registration details or state that the study was not registered. | The secondary analysis was not prospectively registered. | Reported in checklist |
| Open science—Data sharing | 18e | D;E | State data availability. | Raw sequencing data are publicly accessible under CNP0000636 and PRJEB22863; derived QC and analysis results are included in the main and supplementary tables. | Reported |
| Open science—Code sharing | 18f | D;E | State availability of data-processing, feature-engineering, modelling, and evaluation code. | Section 2.10 states that workflows and scripts were version-controlled and frozen; a public repository or formal access route is not specified. | Partly reported |
| Patient/public involvement | 19 | D;E | Describe patient/public involvement or state that none occurred. | No patient or public involvement occurred because this was a secondary analysis of de-identified public datasets. | Reported in checklist |
| Results—Participants | 20a | D;E | Report participant flow, outcome counts, and follow-up; use a diagram where helpful. | Figure 1 and Section 3.1 report screening and exclusions; 41/70 and 23/40 resistance events and PFS follow-up are reported in Table 2. | Reported |
| Results—Participants | 20b | D;E | Report characteristics, dates, predictors, treatment, sample size, events, follow-up, missingness, and demographic differences. | Tables 1-3 and Section 3.1 report clinical characteristics, SMDs, treatments, outcome counts, PFS, sample timing, sequencing/QC, and denominators for missing covariates. | Reported |
| Results—Participants | 20c | E | Compare important predictor and outcome distributions between development and evaluation datasets. | Table 1 compares clinical distributions; Table 3 compares technical characteristics; Figures 2-3 show PBAS-component distributions; between-cohort SMDs and technical heterogeneity are discussed. | Reported |
| Results—Analysis counts | 21 | D;E | Report participant and event counts for each model-development/evaluation analysis. | Counts are reported in Sections 3.1-3.6, Tables 2, 4, and 5, and Figure legends, including complete-case and subgroup analyses. | Reported |
| Results—Model specification | 22 | D | Provide the complete model needed to calculate predictions in new individuals, including access restrictions. | The PBAS feature dictionary and scoring formula are reported, and the logistic form is specified; however, the numerical intercept and full implementation object/code are not presented in the manuscript. | Partly reported |
| Results—Model performance | 23a | D;E | Report performance with uncertainty and useful plots, including key subgroups if examined. | Bootstrap-corrected development performance and external AUC with 95% CI, Brier score, calibration intercept/slope/plot, decision curves, and selected sensitivity/subgroup findings are reported. | Reported |
| Results—Model performance | 23b | D;E | Report heterogeneity in performance across clusters if examined. | No formal cluster-level heterogeneity model was fitted; cohort-specific performance and major between-cohort differences are reported. | Not formally assessed |
| Results—Model updating | 24 | E | Report any updated model and its performance. | No model updating or recalibration was performed. | Not applicable |
| Discussion—Interpretation | 25 | D;E | Interpret main findings in relation to objectives and previous evidence, including fairness considerations. | Discussion interprets the ecological-functional signal, but/buk contrast, prior microbiome studies, exploratory external performance, and limits on subgroup/fairness inference. | Reported |
| Discussion—Limitations | 26 | D;E | Discuss limitations and effects on bias, uncertainty, overfitting, and generalisability. | Limitations address fixed small samples, external imprecision/high PROBAST+AI risk, calibration, missing covariates, residual confounding, antibiotic restriction, technical heterogeneity, unavailable omics, treatment specificity, and overfitting concerns. | Reported |
| Discussion—Usability | 27a | D | Explain how low-quality or missing inputs should be identified and handled in use. | Prespecified QC thresholds and technical exclusions are described; samples failing host depletion, annotation, IGC quantification, or ≥5 million non-host reads are not eligible for score calculation. | Reported |
| Discussion—Usability | 27b | D | Describe required user interaction and expertise. | PBAS calculation requires pretreatment stool shotgun-metagenomic processing and bioinformatic implementation; the manuscript states that it is not an established clinical tool. | Reported |
| Discussion—Next steps | 27c | D;E | Describe next research steps for applicability and generalisability. | Discussion/Conclusion call for larger multicentre prospective validation, standardised technical evaluation, and metabolic/immune-mechanistic studies before clinical translation. | Reported |
